# Mitochondrial and Cell Death Mechanisms in Neurodegenerative Diseases

**DOI:** 10.3390/ph3040839

**Published:** 2010-03-25

**Authors:** Lee J. Martin

**Affiliations:** Department of Pathology, Division of Neuropathology Department of Neuroscience, Johns Hopkins University School of Medicine, 558 Ross Building, 720 Rutland Avenue, Baltimore, Maryland 21205-2196, USA; E-Mail: martinl@jhmi.edu; Tel.: +410-502-5170; Fax: +410-955-9777

**Keywords:** adenine nucleotide translocator, apoptosis, cell death, cyclophilin D, excitotoxicity, fuzzy logic, mitochondrial permeability transition pore, motor neuron, *ppif*, voltage-dependent anion channel

## Abstract

Alzheimer’s disease (AD), Parkinson’s disease (PD) and amyotrophic lateral sclerosis (ALS) are the most common human adult-onset neurodegenerative diseases. They are characterized by prominent age-related neurodegeneration in selectively vulnerable neural systems. Some forms of AD, PD, and ALS are inherited, and genes causing these diseases have been identified. Nevertheless, the mechanisms of the neuronal cell death are unresolved. Morphological, biochemical, genetic, as well as cell and animal model studies reveal that mitochondria could have roles in this neurodegeneration. The functions and properties of mitochondria might render subsets of selectively vulnerable neurons intrinsically susceptible to cellular aging and stress and overlying genetic variations, triggering neurodegeneration according to a cell death matrix theory. In AD, alterations in enzymes involved in oxidative phosphorylation, oxidative damage, and mitochondrial binding of Aβ and amyloid precursor protein have been reported. In PD, mutations in putative mitochondrial proteins have been identified and mitochondrial DNA mutations have been found in neurons in the substantia nigra. In ALS, changes occur in mitochondrial respiratory chain enzymes and mitochondrial cell death proteins. Transgenic mouse models of human neurodegenerative disease are beginning to reveal possible principles governing the biology of selective neuronal vulnerability that implicate mitochondria and the mitochondrial permeability transition pore. This review summarizes how mitochondrial pathobiology might contribute to neuronal death in AD, PD, and ALS and could serve as a target for drug therapy.

## Introduction

Pathologists conceived the concept of cell death as a mechanism of disease to aid in diagnosis and therapy [1]. Pathological stimuli can be extrinsic or intrinsic and can cause abrupt or delayed cell death or inactivate normal cell survival or cell death networks. Many neurological disorders are characterized by undesirable cell death [2], while the absence of precise control of cell number in tissues causes cancer (impaired apoptosis is a central step toward neoplasia) [3]. It is compelling that a goal of human disease management and treatment is, on one hand, to prevent cell death in neurological disease [2] and, on the other hand, to stimulate cell death in malignancy [4]. Thus, the study of cell death is fundamental to human pathobiology and disease mechanisms and the identification of therapeutic targets for disease treatment.

**Figure 1:**
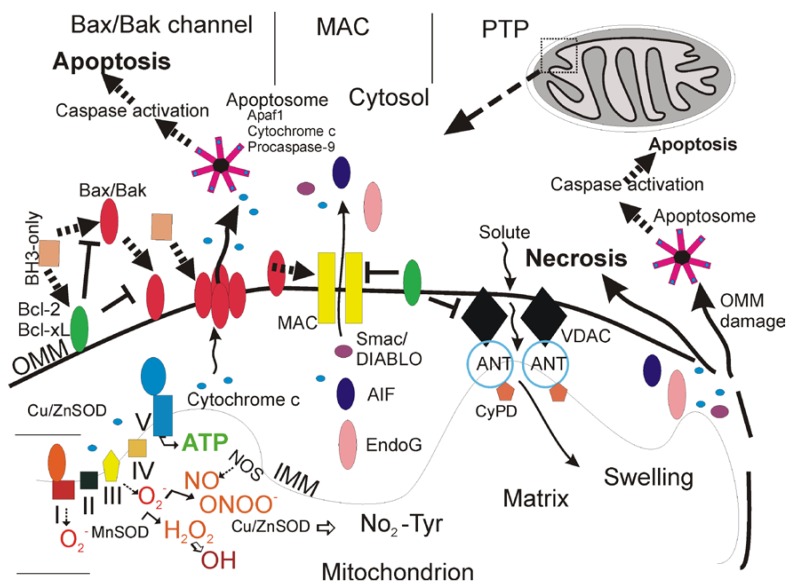
Mitochondria (upper right) are multi-functional organelles and regulate neuronal cell life and death (adapted from an earlier form [5]). See text for descriptions.

An exciting new understanding of mitochondrial biology has emerged over the past two decades from multiple disciplines that is likely to be very relevant to adult-onset neurodegenerative disorders of the central nervous system (CNS). [5]. Mitochondria are multi-functional organelles (Figure 1) [6].

In addition to their critical role in the production of ATP through the electron transport chain (Figure 1), these organelles function in intracellular Ca^2+^ homeostasis, synthesis of steroids, heme and iron-sulfur clusters, and programmed cell death (PCD) [6,7,8]. Mitochondria are also sites of formation of reactive oxygen species (ROS), including superoxide anion (O_2_^•-^) [9] and the highly reactive hydroxyl radical (^•^OH) or its intermediates [10], and reactive nitrogen species such as nitric oxide (^•^NO) [6]. Mitochondria generate endogenous ROS as by-products of oxidative phosphorylation (Figure 1) [8]. Oxygen- and proton pump-driven ATP production by the electron transport chain (Figure 1, lower left) is one function. The respiratory chain proteins (complex I-IV) establish an electrochemical gradient across the inner mitochondrial membrane (IMM) by extruding protons out of the matrix into the intermembrane space, thereby creating an energy gradient that drives the production of ATP by complex V (Figure 1, lower left). Superoxide (O_2_^•-^) is produced as a by-product in the process of electron transport. Electrons in the electron carriers, such as the unpaired electron of ubisemiquinone bound to coenzyme Q binding sites of complexes I, II, and III, can be donated directly to O_2_ to generate O_2_^•-^ [8]. O_2_^•- ^ does not easily pass through biological membranes and thus must be inactivated in compartments where it is generated [9]. The mitochondrial matrix enzyme manganese superoxide dismutase (MnSOD or SOD2) or copper/zinc SOD (Cu/ZnSOD or SOD1) in the mitochondrial intermembrane space and cytosol convert O_2_^•-^ to hydrogen peroxide (H_2_O_2_) in the reaction O_2_^•-^ + O_2_^•-^ + 2H^+^→ H_2_O_2_ + O_2 _ (Figure 1) [9]_. _H_2_O_2 _is more stable than O_2_^•-^ and can diffuse from mitochondria into the cytosol and nucleus. H_2_O_2_ is detoxified by glutathione peroxidase in mitochondria and the cytosol and by catalase in peroxisomes.

Because many mitochondrial proteins possess iron-sulfur clusters for oxidation-reduction reactions and because mitochondrial DNA (mtDNA) lacks protective histones, these macromolecules are particularly vulnerable to ROS attack [8]. In pathological settings that can trigger cell senescence and death, H_2_O_2_ in the presence of reduced transitional metals (Fe^2+^) can be converted to hydroxyl radical (^•^OH) or hydroxyl-like intermediates [10]. O_2_^•-^ can react with the diffusible gas nitric oxide (^•^NO), synthesized by three isoforms of nitric oxide synthase (NOS) enzymes [11], to form the potent nucleophile oxidant and nitrating agent peroxynitrite (ONOO^-^) (Figure 1) [12]. ONOO^- ^or products of ONOO^-^ can damage proteins by nitration [12]. ONOO^- ^is genotoxic directly to neurons by causing single- and double-strand breaks in DNA [13]. Cu/ZnSOD can use ONOO^-^ to catalyze the nitration (NO_2_-Tyr) of mitochondrial protein tyrosine residues (Figure 1, bottom center) such as cyclophilin D (CyPD) and the adenine nucleotide translocator (ANT) which are core components of the mitochondrial permeability transition pore (PTP, another critical function of mitochondria). ^•^NO can be produced in mitochondria [14] and has direct effects in mitochondria. ^•^NO at nanomolar concentrations can inhibit rapidly and reversibly mitochondrial respiration by nitration or nitrosylation [15].

Mitochondrial perturbations are known to participate in the mechanisms of human neuropathology, particularly disorders involving acute interruptions in O_2_ and substrate delivery to the brain and bioenergetic failure as seen in tissue ischemia and toxic exposures [6]. Optic atrophy type 1 (OPA1), a hereditary optic neuropathy, is one example of a chronic neurodegenerative disease caused by mutations in the *OPA1* gene that encodes a mitochondrial dynamin-related GTPase that functions in maintenance of mitochondrial morphology, including fusion, and metabolism [16]. The properties and functions of mitochondria (Figure 1) might confer an intrinsic susceptibility of subsets of long-lived post-mitotic cells such as neurons to aging and stresses, including mutations and environmental toxins. This review summarizes the contributions of the different forms of cell death to three human neurodegenerative diseases (Alzheimer’s disease, Parkinson’s disease, and amyotrophic lateral sclerosis), the evidence for mitochondrial involvement, and their animal and cell models. In this regard varying degrees of mitochondrial dysfunction and intrinsic mitochondrial-mediated cell death mechanisms could be critical determinants in the regulation of disease and neuronal cell death ranging from necrosis and apoptosis to autophagy [17,18,19]; thus, targeting mitochondrial properties or entities, such as the mitochondrial PTP (Figure 1) [20,21,22,23], could be important for developing new mechanism-based pharmaco-therapies for neurodegenerative diseases. 

## Types of Cell Death

**Figure 2:**
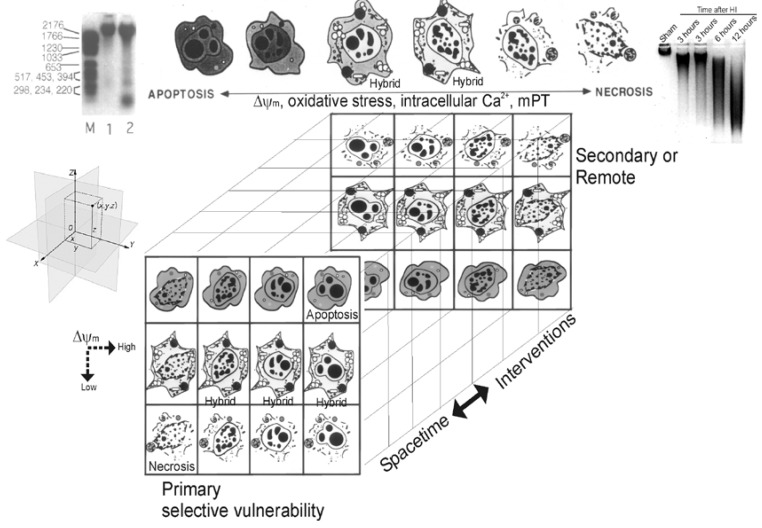
Cell death matrix (modified from its original form [194]). This diagram summarizes in linear (top) and 3-dimensional matrix (bottom) formats the concept of the apoptosis-necrosis continuum of cell death. See text for descriptions.

Cells can die by different processes [24]. These processes have been classified canonically into two distinct categories, called necrosis and apoptosis. These forms of cellular degeneration were classified originally as different because they appeared different morphologically under a microscope (Figure 2).

Necrosis is a lytic destruction of individual or groups of cells, while apoptosis (derived from a Greek word for ‘dropping of leaves from trees’) is an orderly and compartmental dismantling of single cells or groups of cells into consumable components for nearby cells. Apoptosis is an example of programmed cell death (PCD) that is an ATP-driven (sometimes gene transcription-requiring) form of cell suicide often committed by demolition enzymes called caspases, but other apoptotic and non-apoptotic, caspase-independent forms of PCD exist [25]. Apoptotic PCD is instrumental in developmental organogenesis and histogenesis and adult tissue homeostasis, functioning to eliminate excess cells [26]. In healthy people, estimates reveal that between 50 to 70 billion cells in adults and 20 to 30 billion cells in a child between the ages of 8 and 14 die each day due to apoptosis [26]. Another form of cell degeneration is called autophagy [27]. Autophagy is an intracellular catabolic process that occurs by lysosomal degradation of damaged or expendable organelles. Necrosis and apoptosis both differ morphologically (Figure 2) and mechanistically from autophagy [25,27].

More recently the morphological and molecular regulatory distinctions between the different forms of cell death have become blurred and uncertain due to observations made on degenerating neurons in animal models and to a new concept that attempts to accommodate these observations [24,28,29]. This concept, in its original form, posited that cell death exists as a continuum with necrosis and apoptosis at opposite ends of a spectrum with hybrid forms of degeneration manifesting in between (Figure 2) [17,24,28,29]. For example, a hypothetical dying neuron in the CNS is illustrated at coordinates (x,y,z) in the Euclidian coordinate system (Figure 2, at left). The degeneration of this neuron in diseased or damaged animal nervous systems is not always strictly necrosis or apoptosis, according to the traditional binary classification of cell death, but also occurs as intermediate or hybrid forms with coexisting morphological and biochemical characteristics that lie in a structural continuum (Figure 2) [17,24,28,29]. Apoptosis with internucleosomal fragmentation of DNA (Figure 2, top left) and necrosis with random digestion of DNA (Figure 2, top right) are at the extremes and different syncretic hybrid forms are in between (Figure 2, top). The front matrix of the cube shows some of the numerous possible structures of neuronal cell death near or at the terminal stages of degeneration. Combining different nuclear morphologies and cytoplasmic morphologies generates a nonlinear matrix of possible cell death structures. In the cell at the extreme upper right corner (Figure 2), nuclear and cytoplasmic morphologies combine to form an apoptotic neuron that is typical of naturally occurring PCD during nervous system development. This death is classical apoptosis. In contrast, in other cells (Figure 2, extreme lower left corner), the merging of necrotic nuclear and necrotic cytoplasmic morphologies forms a typical necrotic neuron resulting from N-methyl-D-aspartate (NMDA) receptor excitotoxicity and cerebral ischemia (stroke and cardiac arrest). Between these two extremes, hybrids of cell death can be produced with varying contributions of apoptosis and necrosis to the nuclear and cytoplasmic morphologies. Thus, neuronal cell death can be syncretic in a manner similar to that described in fibroblastic cells [30]. The typical apoptosis-necrosis hybrid cell death structure is best exemplified by neurons in the neonatal CNS dying from ischemia or non-NMDA glutamate receptor-mediated excitotoxicity. The death forms shown in the front matrix of the cube represent only a small number of the possible forms of cell death that can be envisioned to fill the empty cells of the matrix. Neuronal maturity and the subtypes of glutamate receptors that are over-activated are known to influence where an injured/degenerating neuron falls within the matrix. The types and levels of DNA damage that are sustained by a cell and concurrent graded activation of mitochondrial permeability transition (mPT) and the levels of oxidative stress and Ca^2+^ might also influence the position of a degenerating cell within the death matrix and in the brain Euclidian coordinate system. The back panel represents the possible cell death forms occurring in space-time over a delayed period after injury or after administration of therapeutic interventions. The matrix predicts that the cell death patterns might change over time from apoptosis to apoptosis-necrosis variants or necrosis and from necrosis to apoptosis-necrosis variants or apoptosis. This prediction could have critical relevance to the idea of neuroprotection. This concept may also be relevant to cell death in general, and thus may be widely applicable to cell biology outside the nervous system.

The *in vivo* reality of a neuronal cell death continuum was revealed first in rat models of glutamate receptor excitotoxicity [28,29]. The hybrid cells can be distinguished cytopathologically by the progressive compaction of the nuclear chromatin into few, discrete, large, irregularly shaped clumps (Figure 2). This morphology contrasts with the formation of few, uniformly shaped, dense, round masses in classic apoptosis and the formation of numerous, smaller, irregularly shaped chromatin clumps in classic necrosis. The cytoplasmic organelle pathology in hybrid cells has a basic pattern that appears more similar to necrosis than to apoptosis but is lower in amplitude than in necrosis (e.g., mitochondrial swelling). Toxicological studies of cultured cells have shown that stimulus intensity influences the mode of cell death [31,32,33], such that apoptosis can be induced by injurious stimuli of lesser amplitude than insults causing necrosis [34], but the cell death modes were still considered distinct [33].

The molecular mechanisms of cell death are becoming known [35,36,37,38], and, with this knowledge, the distinctiveness of the different cell death processes and the potential superposition among different cell death mechanisms are being realized. Experimental studies on cell death mechanisms, and particularly the cell death continuum, are important because they could lead to the rational development of molecular mechanism-based therapies for treating neurodegenerative disorders. The different categories of cell death are discussed below.

### Cell Necrosis and the Mitochondrial Permeability Transition Pore

Cell death caused by cytoplasmic swelling, nuclear dissolution (karyolysis), and lysis has been classified traditionally as necrosis (Figure 2) [39,40]. Cell necrosis (sometimes termed oncosis) [40] can result from rapid and severe failure to sustain cellular homeostasis, notably cell volume control [41]. The process of necrosis involves damage to the structural and functional integrity of the cell plasma membrane and associated enzymes, for example Na^+^,K^+^ ATPase, abrupt influx and overload of ions (e.g., Na^+^ and Ca^2+^) and H_2_O, and rapid mitochondrial damage and bioenergetic collapse [33,42,43,44]. Metabolic inhibition, anoxia, and oxidative stress from ROS can trigger necrosis. Inhibitory crosstalk between ion pumps causes pro-necrotic effects when Na^+^,K^+^ ATPase ‘steals’ ATP from the plasma membrane Ca^2+^ ATPase, contributing to Ca^2+^ overload and mitochondrial damage [45]. 

The morphology and some biochemical features of classic necrosis in neurons are distinctive (Figure 2) [24]. The main features are swelling and vacuolation/vesiculation of organelles, destruction of membrane integrity, digestion of chromatin, and dissolution of the cell. The overall profile of the moribund cell is maintained generally as it degrades into the surrounding tissue parenchyma. The debris instigates an inflammatory reaction in tissue. In necrosis, dying cells do not bud to form discrete, membrane-bound fragments. The nuclear pyknosis and karyolysis appear as condensation of chromatin into many irregularly shaped, small clumps, sharply contrasting with the formation of few, uniformly dense and regularly shaped chromatin aggregates that occurs in apoptosis. In cells undergoing necrosis, genomic DNA is digested globally, because proteases that digest histones, which protect DNA, and DNases are co-activated to generate many randomly sized fragments seen as a DNA ‘smear’ by gel electrophoresis (Figure 2, top right). These cytoplasmic and nuclear changes in pure necrosis are thought to be very diagnostic (Figure 2). 

**Table 1:** Some Mitochondrial Associated Cell Death Proteins.

Protein	Function
Bcl-2*	Anti-apoptotic, blocks Bax/Bak channel formation
Bcl-X_L_	Anti-apoptotic, blocks Bax/Bak channel formation
Bax*	Pro-apoptotic, forms pores for cytochrome *c* release
Bak*	Pro-apoptotic, forms pores for cytochrome *c* release
Bad	Pro-apoptotic, decoy for Bcl-2/Bcl-X_L_ promoting Bax/Bak pore formation
Bid	Pro-apoptotic, decoy for Bcl-2/Bcl-X_L_ promoting Bax/Bak pore formation
Noxa	Pro-apoptotic, decoy for Bcl-2/Bcl-X_L_ promoting Bax/Bak pore formation
Puma	Pro-apoptotic, decoy for Bcl-2/Bcl-X_L_ promoting Bax/Bak pore formation
p53*	Antagonizes activity of Bcl-2/Bcl-X_L_, promotes Bax/Bak oligomerization
Cytochrome *c*	Activator of apoptosome
Smac/DIABLO	IAP inhibitor
AIF	Antioxidant flavoprotein/released from mitochondria to promote nuclear DNA fragmentation
Endonuclease G	Released from mitochondria to promote nuclear DNA fragmentation
HtrA2/Omi	IAP inhibitor
VDAC	mPTP component in outer mitochondrial membrane
ANT^+^	mPTP component in inner mitochondrial membrane
Cyclophilin D^+^	mPTP component in mitochondrial matrix
TSPO (peripheral benzodiazepine receptor)	Modulator of mPTP
Hexokinase	Modulator of VDAC

* Changes have been reported in human ALS (see below) ^+ ^A reported target of oxidative modification in mouse ALS (see below)

Recent work has shown that cell necrosis might not be as chaotic, random, and incomprehensible as envisioned originally but can involve the activation of specific signaling pathways to eventuate in cell death [46,47,48]. This idea is very important for developing new mechanism-based therapeutics to block cell necrosis. For example, DNA damage can lead to poly(ADP-ribose) polymerase activation and ATP depletion, energetic collapse, and necrosis [49]. Other pathways for ‘programmed’ necrosis involve death receptor signaling through NADPH oxidase, receptor-interacting protein 1 (RIP1), and mPT (Figure 1 and Figure 2) [47,48,50,51]. 

mPT is a mitochondrial state in which the proton-motive force is disrupted reversibly or irreversibly [23,50,51,52,53]. Conditions of intra-mitochondrial Ca^2+^ overload, excessive oxidative stress, and decreased electrochemical gradient (Δψ_m_), ADP, and ATP can favor mPT. This alter condition of mitochondria involves the mitochondrial permeability transition pore (mPTP) that functions as a voltage, thiol, and Ca^2+^ sensor [23,50,51,52,53]. The mPTP is believed to be a poly-protein transmembrane channel (Figure 1) formed at the contact sites between the outer mitochondrial membrane (OMM) and inner mitochondrial membrane (IMM). The collective components of the mPTP are still controversial, but the voltage-gated anion channel (VDAC, or porin) in the OMM, the adenine nucleotide translocator (ANT, or solute carrier family 25) in the IMM, and cyclophilin D (CyPD) in the matrix are believed to be the core components (Figure 1 and Table 1) [23,51,53]. Other components or modulators of the mPTP appear to be hexokinase, creatine kinase, translocator protein 18 kDa (TSPO, or peripheral benzodiazepine receptor), and Bcl-2 family members (Table 1) [53].

The VDAC family in human and mouse cells consists of three proteins of ~31 kDa (VDAC1-3) encoded by three different genes [54]. VDACs are the major transport proteins in the OMM, functioning in ATP rationing, Ca^2+^ homeostasis, oxidative stress response, and cell death [54]. Monomeric VDAC serves as the functional channel, although oligomerization of VDAC into dimers and tetramers can occur and might function in cell death [54]. The VDAC adopts an open conformation at low or zero membrane potentials and a closed conformation at potentials above 30–40 mV making the OMM permeable to most small hydrophilic molecules up to 1.3 kDa for free exchange of respiratory chain substrates [55]. Most data implicating VDAC opening or closing as an important regulator of cell death are based on *in vitro* conditions, while limited *in vivo* evidence is available [56]. VDAC1 binds Bcl-2-antagonist/killer 1 (Bak1, see below for description of Bcl-2 family members), hexokinase, gelsolin, and ANT1/ANT2; VDAC2 binds Bak1, hexokinase, cytochrome *c*, glycerol kinase and ANT1/ANT2; VDAC3 binds glycerol kinase, CyPD, and ANT1-3 [54]. In human tissues, VDAC1 and VDAC2 isoforms are expressed more abundantly than VDAC3; highest levels are found in kidney, heart, skeletal muscle, and brain [57]. The effects of selective knockout of VDAC isoforms are not equivalent, implying different functions. Mice deficient in either VDAC1 or VDAC3 are viable [58,59,60], but VDAC2 deficiency causes embryonic lethality [61]. Lack of both VDAC1 and VDAC3 causes growth retardation [60]. VDAC null mouse tissues exhibit deficits in mitochondrial respiration and abnormalities in mitochondrial ultrastructure [58]. However, mitochondria without VDAC1 have an intact mPT response [62,63]. VDAC2 deletion, but not lack of the more abundant VDAC1, results in enhanced activation of the mitochondrial apoptosis pathway and enforced activation of Bak1 at mitochondria [61], consistent with the idea that VDAC2 is a key inhibitor of Bak1-mediated apoptosis [60]. However, other data show that cells lacking individual VDACs or combinations of VDACs have normal death responses to Bax and Bid [63]. Recent work in yeast has revealed that SOD1 is necessary for proper functioning of VDAC; specifically, SOD1 regulates VDAC channel activity and protein levels in mitochondria [64]. 

The mitochondrial ANT family in human consists of 3 members (ANT1-3, or solute carrier family 25, members 4, 5, and 6) encoded by three different genes, but in mouse only two isoforms of the ANT are present [65]. The proteins are ~33 kDa and function as homodimers [65]. They are multi-pass membrane proteins, with odd-numbered transmembrane helices that mediate exchange of cytosolic ADP for mitochondrial ATP across the inner membrane utilizing the electrochemical gradient [66]. These helices have kinks because of proline residues [66]. ANT1 binds VDAC1, CyPD, Bax, twinkle (ataxin-8), and cyclophilin-40; ANT2 binds VDAC1-3 and cyclophilin-40; ANT3 binds VDAC1, steroid sulfatase, and translocase of inner mitochondrial membrane-13 (TIMM13) and TIMM23 [65]. The ANT isoforms are expressed differentially in tissue- and species-specific patterns [67]. ANT1 is expressed highly in human and mouse heart and skeletal muscle; human brain has low ANT1 mRNA but high ANT3 mRNA, while mouse brain has high ANT1 mRNA [67]. ANT2 mRNA is very low or not expressed in most adult human and mouse tissues, except kidney [67]. In tissue mitochondria where more than one ANT isoform is expressed, it is ANT1 that binds preferentially to CyPD to form the mPTP at contact sites between the IMM and OMM (Figure 1) [68]. It has been proposed that, in the presence of high mitochondrial Ca^2+^, the binding of CyPD to proline residue 61 (Pro^61^) in loop 1 of ANT1 results in a conformation that converts the ANT into a non-specific pore [65]. Non-conditional ANT1 null mice are viable and grow normally but develop mitochondrial skeletal myopathy and cardiomyopathy [66]. Ablation both ANT isoforms in mouse liver surprisingly did not change fundamentally mPT and cell death in hepatocytes [69], and some ANT ligands induce mitochondrial dysfunction and cytochrome *c* release independent of mPT [70]. Thus, the mechanisms of ANT-mediated cell deaths need further study.

CyPD (also named cyclophilin F, peptidyl prolyl isomerase F) is encoded by a single gene [23,51,71]. Despite confusing nomenclature, there is only one isoform of CyPD (EC 5.2.1.8, *ppif* gene product) in mouse and human. The ~20 kDa protein encoded by this gene is a member of the peptidyl-prolyl *cis-trans* isomerase (PPIase) family. PPIases catalyze the *cis-trans* isomerization of proline imidic peptide bonds in oligopeptides and accelerate the folding of proteins. CyPD binds ANT1 [66].

During normal mitochondrial function the OMM and the IMM are separated by the intermembrane space, and the VDAC and the ANT do not interact [21,22,23,51]. Permeability transition is activated by the formation of the mPTP (Figure 1); the IMM loses its integrity and the ANT changes its conformation from its native state into a non-selective pore [21,22,23,51]. This process is catalyzed by CyPD functioning as a protein *cis-trans* isomerase and chaperone [20]. The ANT and CyPD interact directly in this process [72]. The amount of CyPD (in heart mitochondria) is much lower than the ANT concentration (< 5%); thus, under normal conditions only a minor fraction of the ANT can be in a complex with CyPD [53,72,73]. When this occurs, small ions and metabolites permeate freely across the IMM and oxidation of metabolites by O_2_ proceeds with electron flux not coupled to proton pumping, resulting in collapse of ΔP, dissipation of ATP production, elevated production of ROS, equilibration of ions between the matrix and cytosol, increased matrix volume, and mitochondrial swelling [52,55]. 

Very few studies have been published on the localizations of mPTP components in the mammalian CNS; thus, details about the cellular expressions in different nervous system cell types are lacking. VDAC expression patterns are complicated by alternative splicing that generates two different VDAC1 mRNAs, three different VDAC2 mRNAs, and two different VDAC3 mRNAs [54]. Studies of nervous tissue have found VDAC in neurons and glial cells [74] and associated with mitochondria, the endoplasmic reticulum (ER), and the plasma membrane [54,76]. Non-mitochondrial localizations of VDAC have been disputed [77]. Information on the cellular localizations of ANT in nervous tissue is scarce. ANT appears to be expressed in reactive astrocytes [78]. The few published studies on CyPD localization in mammalian CNS have found it enriched in subsets of neurons in adult rat brain, with some interneurons being positive [79], and relative low levels in astrocytes [80]. In mouse spinal cord, the core components of the mPTP (VDAC, ANT, and CyPD) are enriched in motor neurons as determined by immunohistochemistry [81]. The specific isoforms of ANT and VDAC in motor neurons have not been determined. CyPD, ANT, and VDAC have mitochondrial and non-mitochondrial localizations in motor neurons [81]. They are all nuclear-encoded mitochondrial-targeted proteins, thus a possible explanation for their non-mitochondrial localizations is that they are pre-mitochondrial forms. Some cyclophilins are located in the cytoplasm [82], such as cyclophilin A, but CyPD immunoreactivity is annulled in *ppif^-/-^* mice, demonstrating that the antibody is detecting only CyPD [81]. Spinal cord, brainstem, and forebrain had similar levels of CyPD, as well as similar levels of ANT and VDAC [81]. Thus, differences in the levels of individual mPTP components cannot explain the intrinsic differences in the sensitivity to Ca^2+^-induced mPT seen in isolated mitochondria from spinal cord and brain [83,84]. Not all mitochondria within individual motor neurons contain CyPD, ANT, and VDAC [81]; this observation supports the idea that mitochondria in individual cells are not only heterogeneous in shape [85,86] but also in biochemical composition, metabolism [87] and genetics [8].

### Apoptosis

Apoptosis is a form of PCD because it is carried out by active, intrinsic transcription-dependent [88] or transcription-independent mechanisms [89] that involve specific molecules (Table 1 and Table 2; Figure 1); the predominance of the different mechanisms in neurons appears to be dictated in part by their maturation state [90]. Kerr and colleagues were the first to describe apoptosis in liver pathology settings [91], but many descriptions cell death different from necrosis were made prior to this time in studies of developing animal systems. Apoptosis should not be used as a synonym for PCD because non-apoptotic forms of PCD exist [92,93]. Apoptosis is only one example of PCD. 

Apoptosis is critical for the normal growth and differentiation of organ systems in vertebrates and invertebrates (see [94] regarding Ernst’s 1926 discovery of developmental PCD) [95,96,97]. In physiological settings in adult tissues, apoptosis is a normal process, occurring continuously in populations of cells that undergo slow proliferation (e.g., liver and adrenal gland) or rapid proliferation (e.g., epithelium of intestinal crypts) [98,99]. Apoptosis is a normal event in the immune system when lymphocyte clones are deleted after an immune response [100]. The structure of apoptosis is similar to the Type I form of PCD described by Clarke [101].

Classical apoptosis has a distinctive structural appearance (Figure 2). The cell condenses and is dismantled in an organized way into small packages that can be consumed by nearby cells. Nuclear breakdown is orderly. The DNA is digested in a specific pattern of internucleosomal fragments (Figure 2), and the chromatin is packaged into sharply delineated, uniformly dense masses that appear as crescents abutting the nuclear envelope or as smooth, round masses within the nucleus (Figure 2). The execution of apoptosis is linked to Ca^2+^-activated DNases [102], one being DNA fragmentation factor 45 (DFF45) [103], that digests genomic DNA at internucleosomal sites only (because proteases that digest histones remain inactivated and the DNA at these sites is protected from DNases) to generate a DNA ‘ladder’ (Figure 2, top left) [102]. However, the emergence of the apoptotic nuclear morphology can be independent of the degradation of chromosomal DNA [104]. Cytoplasmic breakdown during apoptosis is also orderly. The cytoplasm condenses, (as reflected by a darkening of the cell in electron micrographs), and subsequently the cell shrinks in size, while the plasma membrane remains intact. During the course of these events, it is believed that the mitochondria are required for ATP-dependent processes. Subsequently, the nuclear and plasma membranes become convoluted, and, then the cell undergoes a process called budding. In this process, the nucleus, containing smooth, uniform masses of condensed chromatin, undergoes fragmentation in association with the condensed cytoplasm, forming cellular debris (called apoptotic bodies) composed of pieces of nucleus surrounded by cytoplasm with closely packed and apparently intact organelles. Apoptotic cells display surface markers (e.g., phosphatidylserine or sugars) for recognition by phagocyte cells. Phagocytosis of cellular debris by adjacent cells is the final phase of apoptosis *in vivo*.

Variants of classical apoptosis or non-classical apoptosis can occur during nervous system development [101,105] and also frequently in pathophysiological settings of nervous system injury and disease [24,28,29]. Axonal damage (axotomy) and target deprivation in the mature nervous system can induce apoptosis in neurons that is similar structurally, but not identical, to developmental PCD [17]. Excitotoxins and cerebral hypoxia-ischemia can induce readily and robustly non-classical forms of apoptosis in neurons in rodent brain [28,29,106,107,108]. 

Cells can die by PCD through mechanisms that are distinct from apoptosis [92,93]. The structure of non-apoptotic PCD is similar to the Type II or Type III forms of cell death described by Clarke [101]. Interestingly, there is no internucleosomal fragmentation of genomic DNA in some forms of non-apoptotic PCD [92,93].

### Autophagy

Autophagy is a mechanism whereby eukaryotic cells degrade their own cytoplasm and organelles [27]. Autophagy functions as a homeostatic non-lethal stress response mechanism for recycling proteins to protect cells from low supplies of nutrients and as a cell death mechanism. Autophagy is also called Type II PCD [101]. This degradation of organelles and long-lived proteins is carried out by the lysosomal system; thus, a hallmark of autophagy is accumulation of autophagic vacuoles of lysosomal origin. Autophagy has been seen in developmental and pathological conditions. For example, insect metamorphosis involves autophagy [25], and developing neurons can use autophagy as a PCD mechanism [109,110]. Degeneration of Purkinje neurons in the mouse mutant *Lucher* appears to be a form of autophagy, thus linking excitotoxic constitutive activation of the GluRδ2 glutamate receptor to autophagic cell death [110]. However, loss of basal autophagic function in the CNS causes neurodegeneration in mice [112,113]. This finding could be a testimonial to the importance of Parkin, a ubiquitin kinase encoded by PD-related *PARK2*, which functions to promote autophagic turnover of mitochondria [114].

The molecular controls of autophagy appear common in eukaryotic cells from yeast to human, and autophagy may have evolved before apoptosis [35]. However, most of the work has been done on yeast, but detailed work on autophagy in mammalian cells emerging [115]. Double-membrane autophagosomes for sequestration of cytoplasmic components are derived from the ER or the plasma membrane. Tor kinase, phosphatidylinositol 3 (PI3)-kinase, a family of cysteine proteases called autophagins, and death-associated proteins function in autophagy [116,117]. Autophagic and apoptotic cell death pathways crosstalk. The product of the tumor suppressor gene Beclin1 (the human homolog of the yeast autophagy gene APG6) interacts with the anti-apoptosis regulator Bcl-2 [118]. Autophagy can block apoptosis by sequestration of mitochondria. If the capacity for autophagy is reduced, stressed cells die by apoptosis, whereas inhibition or blockade of molecules that function in apoptosis can convert the cell death process into autophagy [119]. Thus, a continuum between autophagy and apoptosis could exist.

## Cellular and Molecular Regulation of Apoptosis

Apoptosis is a structurally and biochemically organized form of cell death (Figure 2). Apoptotic molecular networks are conserved in yeast, hydra, nematode, fruit fly, zebra fish, mouse, and human [120]. The current understanding of the molecular mechanisms of apoptosis in cells is built on studies by Robert Horvitz and colleagues on PCD in a nematode *Caenorhabditis elegans* [121]. They pioneered the understanding of the genetic control of developmental cell death by showing that it is regulated predominantly by three genes (*ced-3, ced-4, and ced-9*) [121]. This seminal work led to the identification of several families of apoptosis-regulation genes (Table 2) in mammals, including the Bcl-2 family [37,38,122] and the caspase family of cysteine-containing, aspartate-specific proteases [123]. Other regulators of apoptotic cell death, most of which are mitochondrial proteins or influence mitochondria, are the p53 gene family, cell surface death receptors, cytochrome *c*, apoptosis inducing factor (AIF), second mitochondrial activator of caspases (Smac), the inhibitor of apoptosis protein (IAP) family, and HtrA2/Omi [100,124,125,126,127,128,129]. 

Specific organelles, including mitochondria and the ER, have been identified as critical for the apoptotic process. In seminal work by Li, Wang, and colleagues, it was discovered that the mitochondrion integrates death signals engaged by proteins in the Bcl-2 family and releases molecules residing in the mitochondrial intermembrane space, such as cytochrome *c*, that complexes with cytoplasmic proteins (e.g., apoptotic protease activating factor 1, Apaf1) to activate caspase proteases leading to internucleosomal cleavage of DNA (Figure 1 and Figure 2) [125,126]. The ER, which regulates intracellular Ca^2+^ levels, participates in a loop with mitochondria to modulate mPT and cytochrome *c* release through the actions of Bcl-2 protein family members (Figure 1) [130].

**Table 2:** Some Molecular Regulators of Apoptosis Relevant to Neurodegeneration and Potential Drug Targeting for Neuroprotection.

**Bcl-2 Family**		**Caspase Family **	**IAP Family**	**Tumor Suppressor **
Anti-apoptotic proteins	Pro-apoptotic proteins			
Bcl-2	Bax	Apoptosis “initiators”: caspase-2, 8, 9, 10	NAIP	p53
Bcl-x_L_	Bak1		Apollon	p63
Mcl-1	Bcl-x_S_		Survivin	p73
Boo/Diva	Bad	Apoptosis “executioners”: caspase-2, 3, 6, 7	IAP1	
	Bid		IAP2	
	Bik		XIAP	
	Bim	Cytokine processors: caspase-1, 4, 5, 11, 12, 14		
	Noxa			
	Puma			

### Bcl-2 family of Cell Survival and Cell Death Proteins

Mitochondria can control cell death (Figure 1) using Bcl-2 family members to regulate apoptosis by modulating the release of cytochrome *c* from mitochondria into the cytosol. Two models can account for this process: the Bcl-2-associated X protein (Bax)/Bak1 channel model and the mitochondrial apoptosis-induced channel or MAC) (Figure 1). The *bcl-2* proto-oncogene family is a large group of apoptosis regulatory genes encoding about 20 different proteins. These proteins are defined by at least one of four conserved B-cell lymphoma (Bcl) homology domains (BH1-BH4) in their amino acid sequence that function in protein-protein interactions [37,38,122]. Some of the proteins (e.g., Bcl-2, Bcl-x_L_, and Mcl-1) have all four BH1-BH4 domains and are anti-apoptotic (Table 2). Other proteins that are pro-apoptotic have BH1-BH3 sequences (e.g., Bax and Bak1) or only the BH3 domain (e.g., Bad, Bid, Bim, Bik, Noxa, and Puma) that contains the critical death domain (Table 2). Bcl-x_L_ and Bax have α-helices resembling the pore-forming subunit of diphtheria toxin [131]; thus, Bcl-2 family members appear to function by conformation-induced insertion into the outer mitochondrial membrane to form channels or pores that can regulate release apoptogenic factors (Figure 1). Bcl-2 family members can form homodimers or heterodimers and higher-order multimers with other family members. Bax/Bak1 heterodimerization with either Bcl-2 or Bcl-x_L_ neutralizes their pro-apoptotic activity. When Bax and Bak1 are present in excess, the anti-apoptotic activity of Bcl-2 and Bcl-x_L_ is antagonized, and apoptosis is promoted. 

The expression of many of these proteins is regulated developmentally, and they have differential tissue distributions and subcellular localizations. Most of these proteins are found in CNS. The subcellular distributions of Bax, Bak1, and Bad in healthy adult rodent CNS tissue [132] are consistent with what is known about these proteins in cultured mammalian non-neuronal cells [133,134]. Bax, Bad, and Bcl-2 reside primarily in the cytosol, whereas Bak1 resides primarily in mitochondria. 

Release of cytochrome *c* from mitochondria (Figure 1) can occur through mechanisms that involve the formation of membrane channels comprised of Bax or Bak1 [135] and Bax and the VDAC [136]. In the Bax/Bak1 channel model (Figure 1, left), after specific cell death inducing stimuli Bax undergoes a conformation shift and translocates to the OMM where it inserts. Bak1 is a similar pro-apoptotic protein localized mostly to the OMM. Bax/Bak1 monomers physically interact to form oligomeric or heteromeric channels that are permeable to cytochrome *c*. The formation of these channels is blocked by Bcl-2 and Bcl-x_L_ at multiple sites. BH3-only members (Bad, Bid, Noxa, Puma) are pro-apoptotic and can modulate the conformation of Bax/Bak1 to sensitize this channel, possibly by exposing its membrane insertion domain. The MAC could be a channel similar to the Bax/Bak1 channel, but it might also have additional components such as VDAC. 

Released cytochrome *c* then triggers the assembly of the cytoplasmic apoptosome. The apoptosime is a protein complex of apoptotic protease activating factor 1 (Apaf1), cytochrome *c*, and procaspase-9; this is the engine that drives caspase-3 activation in mammalian cells (Figure 1) [125]. Bcl-2 and Bcl-x_L_ block the release of cytochrome *c* [137,138] from mitochondria and thus the activation of caspase-3 (Figure 1) [125,126]. This Bcl-2 and Bcl-x_L _mediated retention of mitochondrial cytochrome *c* [126,139] is caused by inhibition of Bax channel-forming activity in the outer mitochondrial membrane [135] or by modulation mitochondrial membrane potential and volume homeostasis [139]. BH3-only proteins such as Bim, Bid, Puma, and Noxa appear to induce a conformational change in Bax or they serve as decoys for Bcl-x_L _that allow Bax to form pores in the outer mitochondrial membrane [140]. Cells without *bax* and *bak* genes are resistant to mitochondrial cytochrome *c* release during apoptosis [141]. 

Some anti-apoptotic proteins also have functions downstream of mitochondria. For, example Bcl-x_L_ has anti-apoptotic activity by interacting with Apaf1 and caspase-9 and inhibiting Apaf1-mediated autocatalytic maturation of caspase-9 [142]. Boo can inhibit Bak- and Bik-induced apoptosis (but not Bax-induced cell death) possibly through heterodimerization and by interactions with Apaf1 and caspase-9 [143]. 

Protein phosphorylation regulates the functions of some Bcl-2 family members having downstream mitochondrial consequences. Bcl-2 loses its anti-apoptotic activity following serine phosphorylation, possibly because its antioxidant function is inactivated [144]. Bcl-2 can also associate with non-homologous proteins, including the protein kinase Raf-1 [145]. This association can target Raf-1 to mitochondrial membranes, allowing this kinase to phosphorylate Bad at serine residues [145]. The phosphatidylinositol 3-kinase (PI3-K) –Akt pathway also regulates the function of Bad [146,147] and caspase-9 [148] through phosphorylation. In the presence of sufficient trophic factors, Bad is phosphorylated. Phosphorylated Bad is sequestered in the cytosol by interacting with soluble protein 14-3-3 and, when bound to protein 14-3-3, Bad is unable to interact with Bcl-2 and Bcl-x_L_, thereby promoting cell survival [149]. Conversely, when Bad is dephosphorylated by calcineurin [150], it dissociates from protein 14-3-3 in the cytosol and translocates to the mitochondria where it exerts pro-apoptotic activity. Non-phosphorylated Bad heterodimerizes with membrane-associated Bcl-2 or Bcl-x_L _to liberate Bax from Bax-Bcl-2 and Bax-Bcl-X_L_ dimers, thus promoting cell death [109]. In liver mitochondria, Bad and glucokinase exist in a complex that functions in mitochondrial-based glucokinase activity and mitochondrial respiration in response to glucose [152]. Glucose deprivation results Bad dephosphorylation and Bad-dependent cell death, thereby linking glucose metabolism to apoptosis [152]. 

### Caspase Family of Cell Demolition Proteases

Caspases (*c*ysteinyl *asp*artate-specific protein*ases*) are cysteine proteases that have a near absolute substrate requirement for aspartate in the P_1_ position of the peptide bond. Fourteen *caspase* genes have been identified in mammals [153]. Some caspases (e.g., caspase-12) in human and mouse function differently and have different contributions to cell death mechanisms. Caspases exist as constitutively expressed inactive pro-enzymes (30–50 kDa) in healthy cells. Caspase zymogens are found in different proportions at different subcellular locations. In HeLa cells, most caspase-3 pro-enzyme is found in the cytoplasm, while only 10% is found in mitochondria [154]. In rat heart and brain, 90% of caspase-9 pro-enzyme is mitochondrial [155]. The zymogens contain 3 domains: an amino-terminal pro-domain; a large subunit (~20 kDa); and a small subunit (~10 kDa). Caspases are activated through regulated proteolysis of pro-enzyme with “initiator” caspases activating “executioner” caspases (Table 1; Figure 1). Other caspase family members function in inflammation by processing cytokines (Table 1) [153]. 

The pro-domain of initiator caspases contains amino acid sequences that are caspase recruitment domains (CARD) or death effector domains (DED) that enable the caspases to interact with other molecules that regulate their activation. Activation of caspases involves proteolytic processing between domains, and then association of large and small subunits to form a heterodimer with both subunits contributing to the catalytic site. Two heterodimers associate to form a tetramer that has 2 catalytic sites that function independently. Some isoforms of caspases (e.g., caspase-9, isoform 2) are inactive proteolytically and function as dominant negative inhibitors of active forms.

Active caspases have many target proteins [112] that are cleaved during regulated and organized cell death. Caspases cleave nuclear proteins (e.g., DNases, poly(ADP) ribose polymerase, DNA-dependent protein kinase, heteronuclear ribonucleoproteins, transcription factors or lamins), cytoskeletal proteins (e.g., actin and fodrin), and cytosolic proteins (e.g., other caspases, protein kinases, Bid). 

In human cell line models of apoptosis (Figure 1), activation of caspase-3 occurs when caspase-9 pro-enzyme (also known as Apaf3) is bound by Apaf1 that then oligomerizes in a process initiated by cytochrome *c* (identified as Apaf2) and either ATP or dATP [125]. Cytosolic ATP or dATP are required cofactors for cytochrome *c*-induced caspase activation [125]. Apaf1, a 130 kDa cytoplasmic protein, serves as a docking protein for procaspase-9 and cytochrome *c* [125]. Apaf1 becomes activated when ATP is bound and hydrolyzed, with the hydrolysis of ATP and the binding of cytochrome *c* promoting Apaf1 oligomerization [113]. This oligomeric complex recruits procaspase-9 (forming the apoptosome) and mediates the autocatalytic activation of caspase-9 that disassociates from the complex and becomes available to activate caspase-3 (Figure 1). Once activated, caspase-3 cleaves a protein with DNase activity (*i.e.*, DFF-45), and this cleavage activates a process leading to the internucleosomal fragmentation of genomic DNA (Figure 2, top left) [103].

So far three caspase-related signaling pathways have been identified that can lead to apoptosis [103,125,126,157], but crosstalk among these pathways is possible. The intrinsic mitochondria-mediated pathway is controlled by Bcl-2 family proteins. It is regulated by cytochrome *c* release from mitochondria, promoting the activation of caspase-9 through Apaf1 and then caspase-3 activation. The extrinsic death receptor pathway involves the activation of cell-surface death receptors (see below), including Fas and tumor necrosis factor receptor, leading to the formation of the death-inducing signaling complex (DISC) and caspase-8 activation that in turn cleaves and activates downstream caspases such as caspase-3, -6, and -7. Caspase-8 can also cleave Bid leading to the translocation, oligomerization, and insertion of Bax or Bak1 into the mitochondrial membrane. Another pathway involves the activation of caspase-2 by DNA damage or ER stress as a pre-mitochondrial signal [158]. 

### Inhibitor of Apoptosis Protein (IAP) Family

The activity of pro-apoptotic proteins is blocked to prevent untimely apoptosis in normal cells. Apoptosis can be antagonized by the IAP family in mammalian cells [159,160,161]. This family includes X chromosome-linked IAP (XIAP), IAP1, IAP2, neuronal apoptosis inhibitory protein (NAIP), Survivin, Livin, and Apollon. These proteins are characterized by 1 to 3 baculoviral IAP repeat domains consisting of a zinc finger domain of ~70–80 amino acids [160]. Apollon is a huge (530 kDa) protein that also has a ubiquitin-conjugating enzyme domain. The main identified anti-apoptotic function of IAPs is the suppression of caspase activity [161]. Procaspase-9 and procaspase-3 are major targets of several IAPs. IAPs reversibly interact directly with caspases to block substrate cleavage. Apollon also ubiquitinates and facilitates proteasomal degradation of active caspase-9 and second mitochondria-derived activator of caspases (Smac) [162]. However, IAPs do not prevent caspase-8-induced proteolytic activation of procaspase-3. IAPs can also block apoptosis by reciprocal interactions with the nuclear transcription factor NFκB [160]. 

Scant information is available on IAPs in the nervous system. Survivin is essential for nervous system development in mouse because conditional deletion of *survivin* gene in neuronal precursor cells causes reduced brain size and severe multifocal degeneration and death shortly after birth [163]. NAIP is expressed throughout the CNS in neurons [164]. XIAP is enriched highly in mouse spinal motor neurons [165]. The importance of the *IAP* gene family in pediatric neurodegeneration is underscored by the finding that NAIP is deleted partially in a significant proportion of children with spinal muscular atrophy [166].

Mitochondrial proteins exist that inhibit mammalian IAPs. A murine mitochondrial protein called Smac and its human ortholog DIABLO (for direct IAP-binding protein with low pI) inactivate the anti-apoptotic actions of IAPs and thus exert pro-apoptotic actions [167,168]. Smac/DIABLO are released into the cytosol to inactivate the anti-apoptotic actions of inhibitor of apoptosis proteins that inhibit caspases (Figure 1). These IAP inhibitors are 23 kDa mitochondrial proteins (derived from 29 kDa precursor proteins processed in the mitochondria) that are released into the cytosol from the intermembrane space to sequester IAPs. High temperature requirement protein A2 (HtrA2), also called Omi, is another mitochondrial serine protease that exerts pro-apoptotic activity by inhibiting IAPs [169]. HtrA2/Omi functions as a homotrimeric protein that cleaves IAPs irreversibly, thus facilitating caspase activity. The intrinsic mitochondrial-mediated cell death pathway is regulated by Smac and HtrA2/Omi [169]. Mutations in the *htra2* gene, identified as *PARK13* (Table 3), have been linked to the development of Parkinson’s disease [170], but this linkage is controversial [171]. 

### Apoptosis Inducing Factor (AIF)

AIF is a mammalian cell mitochondrial protein identified as a flavoprotein oxidoreductase [172]. AIF has an N-terminal mitochondrial localization signal that is cleaved off to generate a mature protein of 57 kDa after import into the inter-mitochondrial membrane space. Under normal physiological conditions AIF might function as a ROS scavenger targeting H_2_O_2_ [127] or in redox cycling with nicotinamide adenine dinucleotide phosphate [173]. After some apoptotic stimuli, AIF is released from mitochondria (Figure 1) and translocates to the nucleus [172]. Over-expression of AIF in cultured cells induces cardinal features of apoptosis, including chromatin condensation, high molecular weight DNA fragmentation, and loss of mitochondrial transmembrane potential [172].

### p53/p63/p73 Family of Tumor Suppressors

Cell death by apoptosis can be triggered by DNA damage. p53 and related DNA binding proteins identified as p73 and p63 are involved in this process [124]. p53, p73 and p63 function in apoptosis as well as growth arrest and repair. They can commit to death cells that have sustained DNA damage from ROS, irradiation, and other genotoxic stresses [124]. p53 and p73 have similar oligomerization and DNA sequence transactivation properties. p73 exists as a group of full-length isoforms (including p73α and p73β) and as truncated isoforms that lack the transactivation domain (∆N-p73). p53 is the most well studied of this family of proteins.

p53 is a short-lived protein with a half-life of ~5-20 min in most types of cells studied but can rapidly accumulate several-fold in response to DNA damage. This rapid regulation is mediated by posttranslational modification such as phosphorylation and acetylation as well as intracellular redox state [174]. The elevation in p53 protein levels occurs through stabilization and prevention of degradation. p53 is degraded rapidly in a ubiquitination-dependent proteasomal pathway [175]. Murine double minute 2 (Mdm2, the human homolog is Hdm2) has a crucial role in this degradation pathway [176]. Mdm2 functions in a feedback loop to limit the duration or magnitude of the p53 response to DNA damage. Expression of the *mdm2* gene is controlled by p53 [176]. Mdm2 binds to the N-terminal transcriptional activation domain of p53 and regulates its DNA binding activity and stability by direct association. Mdm2 has ubiquitin ligase activity for p53 through the ubiquitin-conjugating enzyme E2. Stabilization of p53 is achieved through phosphorylation of serine^15^ resulting in inhibition of formation of Mdm2-p53 complexes. Activated p53 binds the promoters of several genes encoding proteins associated with growth control and cell cycle checkpoints (e.g., p21, growth-arrest and DNA damage-45, Mdm2) and apoptosis (e.g., Bax, Bcl-2, Bcl-x_L_, and Fas). The BH3-only proteins Puma and Noxa are critical mediators of p53-mediated apoptosis [137].

p53 can mediate cell death through extra-nuclear transcriptional-independent mechanisms. p53 can translocate rapidly to mitochondria in response to genotoxic, hypoxic, and oxidative stresses in non-neuronal cells [178] and in neurons [90]. This localization can mediate mitochondrial membrane permeabilization through direct physical interaction with Bax [179] and activation of Bak through disruption of the Bak-Mcl1 complex [180]. 

p53 can drive apoptosis in cultured sympathetic ganglion neurons in response to neurotrophin withdrawal [181] and in cultured mouse cortical neurons in response to DNA damage [90]. A small-molecule inhibitor of p53 binding to mitochondria protects against neuronal apoptosis in cultured mouse cortical neurons [90]. p53-mediated neuronal apoptosis *in vitro* can be blocked by the ∆N-p73 isoform by direct binding and inactivation of p53 [141]. *In vivo* experiments show that *p53* gene ablation protects against neuronal apoptosis induced by axotomy and target deprivation in rodent brain and spinal cord [182,183]. 

### Cell Surface Death Receptors

Cell death can also be initiated at the cell membrane by surface death receptors of the tumor necrosis factor (TNF) receptor family. Fas (CD95/Apo-1) and the 75-kDa neurotrophin receptor (p75^NTR^) are members of the large TNF receptor family [100]. Signals for apoptosis are initiated at the cell surface by aggregation (trimerization) of the death domain containing members of this receptor family by their specific ligand. Fas death receptor-mediated apoptosis is a well described pathway for death receptor signaling and is independent of new RNA or protein synthesis. Activation of Fas is induced by binding of the multivalent Fas ligand (FasL), a member of the TNF-cytokine family. FasL is expressed on activated T cells and natural killer cells. Clustering of Fas on the target cell by FasL recruits Fas-associated death domain (FADD), a cytoplasmic adapter molecule that functions in the activation of the caspase 8-Bid pathway, thus forming the DISC [185]. Signaling for apoptosis then proceeds via the extrinsic or intrinsic pathway. In the extrinsic pathway, active caspase-8 then directly cleaves caspase-3 [100]. Activation of the mitochondrial or intrinsic pathway proceeds via caspase 8 mediated cleavage of cytosolic Bid [185]. The truncated form of Bid then translocates to mitochondria, thereby functioning as a BH3-only transducer of Fas activation signal at the cell plasma membrane to mitochondria [185]. Bid translocation from the cytosol to mitochondrial membranes is associated with a conformational change in Bax (that is prevented by Bcl-2 and Bcl-x_L_) and is accompanied by release of cytochrome *c* from mitochondria [186]. 

Apoptosis can also be mediated by p75^NTR ^[187]. Activation of p75^NTR ^occurs by binding of nerve growth factor. When p75^NTR ^is activated without tropomyosin receptor kinases, neurotrophin binding induces homodimer formation and generation of ceramide through sphingomyelin hydrolysis. Ceramide production is associated with the activation of Jun N-terminal kinase (JNK) that phosphorylates and activates c-Jun and other transcription factors. p75^NTR ^mediates hippocampal neuron death in response to neurotrophin withdrawal, involving cytochrome *c*, Apaf1, and caspases-9, -6, and -3 (but not caspase-8), and thus is different from Fas-mediated cell death [187].

Evidence for the importance of these signaling pathways in experimental brain injury is growing. Activation of multiple components of the Fas death receptor signaling pathway have been found in rat and mouse models of motor neuron degeneration [188] and blocking Fas death receptor signaling by genetic means affords protection in these models [188]. Neuron degeneration caused by target deprivation *in vivo* appears to be driven in part by a death receptor-dependent pathway [18]. 

## Excitotoxic Neuronal Cell Death

Neuronal death can be induced by excitotoxicity. This observation was made originally by Lucas and Newhouse in 1957 [189], formulated into a concept by John Olney after showing that glutamate can kill neurons in brain [190], and then examined mechanistically by Dennis Choi, Steven Rothman and others [191,192]. This concept has fundamental importance to neural mitochondria pathobiology and to a variety of acute neurological insults, such as cerebral ischemia and trauma, and possibly chronic neurodegenerative diseases [2,7,17,24,33,43,193,194,195]. Excitotoxicity is pathologic neurodegeneration mediated by excessive activation of glutamate-gated ion channel receptors and voltage-dependent ion channels. Increased cytosolic free Ca^2+^ causes activation of Ca^2+^-sensitive proteases, protein kinases/phosphatases, phospholipases, and NOS when glutamate receptors are stimulated. The excessive interaction of ligand with subtypes of glutamate receptors causes pathophysiological changes in intracellular ion concentrations, pH, protein phosphorylation, energy metabolism, and mitochondrial function and movement [194,195]. Intracellular Ca^2+^ elevations can halt anterograde movement of mitochondria through kinesin-1 and the atypical GTPase Miro [196]. The mPTP is also involved at least in cell culture models of excitotoxicity [195]. The precise mechanisms of excitotoxic cell death and its relationships to mitochondria are still being examined intensively, driven by the hope of identifying therapeutic targets for neurological/neurodegenerative disorders with putative excitotoxic components. Evidence that the uncompetitive, low-affinity, NMDA receptor open-channel blocker shows benefits in AD and other human neurodegenerative diseases [196] justifies continued work on the excitotoxicity concept, but other mechanisms need to be folded into excitotoxicity theory, including sodium-calcium exchangers, volume-regulated anion channels, and acid-sensing channels, to complete the concept. A more complete excitotoxicity theory might help to validate the cell death matrix concept (see below) and to explain why cell culture and animal experimental data are discordant with regard to whether excitotoxic neuronal death is apoptosis, necrosis, apoptosis-necrosis hybrids, autophagy, or perhaps even a peculiar form of cell death that is unique to excitotoxicity. 

The contribution of apoptotic mechanisms to excitotoxic death of neurons has been examined in cultured neurons. Excitotoxicity can cause activation of endonucleases and specific internucleosomal DNA fragmentation in cultures of cortical neurons [197,198] and cerebellar granule cells [199,200]. Internucleosomal fragmentation of DNA was not observed in other experiments on cerebellar granule cell cultures [201]. Excitotoxic cell death in neuronal cultures is prevented [198] or unaffected [197,200,201] by inhibitors of RNA or protein synthesis and is sensitive [198,200] or insensitive [201] to the endonuclease inhibitor aurintricarboxylic acid. In primary cultures of mouse cortical cells, the non-NMDA glutamate receptor agonist kainic acid (KA) induces increased Bax protein, and *bax* gene ablation significantly protects cells against KA receptor toxicity [202]. However, NMDA receptor toxicity in mouse cerebellar granule cells [203] and mouse cortical cells [204] was not Bax-dependent. These results suggest that non-NMDA glutamate receptor excitotoxicity is more likely than NMDA receptor-mediated excitotoxicity to induce apoptosis or apoptosis-necrosis hybrid cell death [17,28,29]; however, species-specific responses might be operative. Glutamate (100 μM) stimulation of mouse cortical cells did not cause an increase in caspase activity [205], but NMDA treated rat cortical cells showed increased caspase activity [206]. In cerebellar granule neurons, glutamate (100 µM - 1 mM) did not activate caspase activity and adenoviral-mediated expression of IAPs did not influence excitotoxic cell death [207]. These conflicting results can also be related to the finding that activation of different subtypes of glutamate receptors appears to engage different modes of cell death [17,28,29]. 

The precise mechanisms of excitotoxic neuronal cell death in animals have not been identified. The morphology of excitotoxicity in many neurons in rodents and large animals injected intracerebrally with excitotoxins include somatodendritic swelling, mitochondrial damage, and chromatin condensation into irregular clumps (Figure 2) [17,28,29,190,208], features that are thought to be typical of cellular necrosis; however, in other neurons, excitotoxicity causes cytological features more like apoptosis [28,29,208]. Excitotoxic degeneration of hippocampal CA3 neurons in response to KA is increased in *naip* gene-deleted mice, supporting a contribution of capsase-dependent apoptosis [209]. Excitotoxic neurodegeneration in adult rat brain has been shown to be either sensitive [160] or insensitive [211] to protein synthesis inhibition. 

In newborn rodents, injection of KA or the NMDA receptor agonist quniolinic acid into the forebrain causes copious apoptosis of cortical, hippocampal, and striatal neurons serving as models of apoptosis in neurons *in vivo* [212,213]. This apoptosis has been verified structurally with light microscopy and EM and by immunolocalization of cleaved caspase-3 [212,123]. Ubiquitous apoptosis is observed at 24 hours after the insult. DNA degradation by internucleosomal fragmentation further confirms the presence of apoptosis. Excitotoxic neuronal apoptosis is associated with rapid (within 2 hours after neurotoxin exposure) translocation of Bax and cleaved caspase-3 to mitochondria [212]. 

A study has revealed that the ratio of mitochondrial membrane-associated Bax to soluble Bax in normal developing striatum changes prominently with brain maturation [212]. Newborn rat striatum has a much greater proportion of Bax in the mitochondrial fraction relative to soluble Bax [212]. Mature rat striatum has a much larger proportion of Bax in the soluble fraction relative to Bax in the mitochondrial fraction [212]. With brain maturation there is a linear decrease in the ratio of mitochondrial Bax to soluble Bax [212]. This developmental subcellular redistribution of Bax might be a reason why immature rodent neurons exhibit a more robust classical apoptosis response compared to adult neurons after brain damage [212]. 

## The Cell Death Continuum

Animal models of neurodegeneration have revealed that age or maturity of brain and the subtype of excitatory glutamate receptor that is activated appear to influence the cytological features and rate of neuronal cell death [17,24,28,29,212,213,214]. This structural and temporal diversity of neuronal cell death is seen with a variety of brain injuries including excitotoxicity, cerebral hypoxia-ischemia, target deprivation, and axonal trauma. Hence, injury-associated neuronal death is not the same in immature and mature CNS and can be pleiomorphic in neurons within the same brain (Figure 2). 

To help explain these data the concept of the cell death continuum was formulated (Figure 2). In this concept cell death exists as a continuum of necrosis and apoptosis with numerous hybrid forms of degeneration manifesting between necrosis and apoptosis (Figure 2) [17,24,28,29]. A fundamental cornerstone of the cell death continuum concept is thought to be gradations in the responses of cells to stress, particularly gradations in mitochondrial dysfunction and mPTP activation [108,215]. Some specific mechanisms thought to drive the continuum are the developmental expression of different subtypes of glutamate receptors, mitochondrial bioenergetics and membrane protein composition (e.g., Bax and mPTP components), the propinquity of developing neurons to the cell cycle, neurotrophin requirements and extensiveness of axonal collateralization, DNA damage vulnerability, and DNA repair mechanism availability [216]. Although the molecular mechanisms that drive this cell death continuum in the intact CNS are uncertain currently, cell culture data hint that ATP levels [42], intracellular Ca^2+^ levels [36] and mPT [50,51] could be involved. Whole animal experiments suggest that the relative level of Bax in the OMM could regulate the cell death continuum in neurons [212]. 

The concept of the cell death continuum has been challenged and deemed as confusing [217,218,219]. Do morphology and underlying biochemical processes of cell death remain binary and discrete [217]? While this is the case at the extremes of the cell death continuum, absolute discreteness ignores the observable features of cell degeneration seen in the injured and diseased CNS. Cell death is more than binary, it is multi-valued. Other possibilities that might have bearing on the reality of the cell death continuum concept include: 1) excitotoxic neuronal death *in vivo* is necrotic, regardless of age, and 2) apoptosis of neurons in the adult nervous system is extremely infrequent [217]. Experiments done by us [17,28,29,106,107,108] and others [220,221,222,223] have shown that neuronal degeneration triggered by excitotoxicity and hypoxia-ischemia can be apoptosis, apoptosis-necrosis hybrids, necrosis, and autophagy; furthermore, entire populations of neurons in the adult rodent CNS can indeed undergo apoptosis after injury [182,183]. 

Rigid conceptualization of cellular pathology is not realistic and is misleading and can hinder the goal of identification of relevant molecular mechanisms of neurodegeneration in complex biological systems, such as the developing, ageing or injured CNS, and ultimately limit the realization of therapeutic opportunities. For example, motor neuron degeneration in amyotrophic lateral sclerosis (ALS) was not considered to be a variant of apoptosis until the concept of the cell death continuum was applied [224], and now anti-apoptosis therapies are in clinical trials for the treatment of ALS [225]. The concept of the cell death continuum (Figure 2) might also be applicable to cytopathology in general, when dealing with cells that are resistant to one form of cell death. 

## The Cell Death Matrix

Studies show that morphologic appearance of the dying cell is a valuable tool for providing hints about the biochemical and molecular events responsible for the cell death type [17,18]. When studying mechanisms of cell death in human disease and in animal and cell models of disease it can be helpful to embrace the idea that apoptosis, necrosis, autophagy, or non-apoptotic PCD are not strictly “black and white”. For the nervous system, overlay this complexity with cell death mechanisms that are influenced by brain maturity, post-mitotic state of neurons, capacities for protein/RNA synthesis and DNA repair, antioxidant/redox status, neurotrophin requirements, death receptor expression, location in brain and location relative to the primary sites of injury, as well as intensity of the insult and mPT (Figure 2). 

To help better comprehend neurodegeneration and discover laws that determine causes and effects in neurodegenerative settings, the concept of the cell death continuum was extended to a hypothetical cell death matrix to embrace the ‘fuzziness’ of cell death in the injured CNS (Figure 2). A matrix might be a useful modeling tool for pathology in general and specifically for predicting the contributions of the different forms of cell death, and the possible identification of previously unrecognized forms of cell death in human neurological disorders and in their animal/cell models. The cell death matrix draws on the framework of biological space-time. It integrates space (location in brain, location of primary insult) and time into a continuum; thus, cell death manifests in a brain regional 3-dimensional context with time playing the role of a fourth dimension that is of a different context than the spatial dimension. By combining space and time into a single matrix we organize a large number of cell death phenotypes and potential mechanisms into a manageable frame of reference to reveal the potential early and delayed responses of the brain to stress/injury and therapeutic interventions.

We need to identify better the relationships between mechanisms of cell death and the structure of dying cells in human pathology, in developing and adult CNS, as well as in animal and cell models of neurotoxicity in undifferentiated immature and terminally differentiated cells. The concept of a cell death matrix could be important for understanding neuronal degeneration in a variety of pathophysiological settings, and thus may be important for mechanism-based neuroprotective treatments in neurological disorders in infants, children, and adults. If brain maturity and brain location dictate how and when neurons die relative to the insult [17,18], then the molecular mechanisms responsible for neuronal degeneration in different brain regions (and at different times after the injury) in infants and children might be different from the mechanisms of neuronal degeneration in adults; hence, therapeutic targets will differ, and, thus, therapies will need to be customized for different brain regions, post-insult times, and age groups.

A cell death matrix could also be useful for modeling outcomes and how drugs and other treatments for human disease will work. It will be extremely important to use clues from cell death structure following different types and degrees of brain injury to better understand which injuries are most likely to respond to anti-necrosis, anti-apoptosis, or combination therapies and whether these therapies actually ameliorate injury or simply delay or change the mode of cell death. We predict that apoptosis inhibitors alone will be inadequate to ameliorate neurodegeneration in most settings, because if the cell death continuum is real, then apoptosis inhibitor drugs could simply push cell degeneration from apoptosis to apoptosis-variant, autophagy, or necrotic cell death as seen in cultured fibroblastic cells treated with caspase inhibitors after chemical hypoxia [30]. Using the cell death matrix we predict that it will be difficult to pinpoint appropriate times for effective mechanism-based, spatially-directed drug therapy for neuroprotection.

## Cell Death in Human Neurodegenerative Diseases

### Alzheimer’s disease (AD)

AD is the most common cause of dementia occurring in middle and late life [226]. Population based surveys estimate that AD affects 7–10% of individuals >65 years of age and possibly 50–60% of people over 85 years of age [227,228]. AD now affects about 2% of the population, or about 4 million people in the USA and ~35 million people worldwide [229]. The prevalence of AD is increasing proportionally to increased life expectancy and estimates predict that the prevalence will reach ~107 million by 2050 [230]. 

Most cases of AD have unknown etiologies and are called sporadic and have a late onset; however, some cases, particularly those with early onset, are familial and are inherited as autosomal dominant disorders linked to mutations in the gene that encodes amyloid precursor protein (APP) [231,232,233,234] or genes that encode for presenilin proteins [235,236]. For late onset sporadic cases, a variety of risk factors have been identified in addition to old age [237]. The apolipoprotein E (ApoE) allele is a susceptibility locus with the ApoE4 type showing dose-dependent contributions [238]. Cardiovascular disease and head trauma are additional risk factors for AD [226].

**Figure 3:**
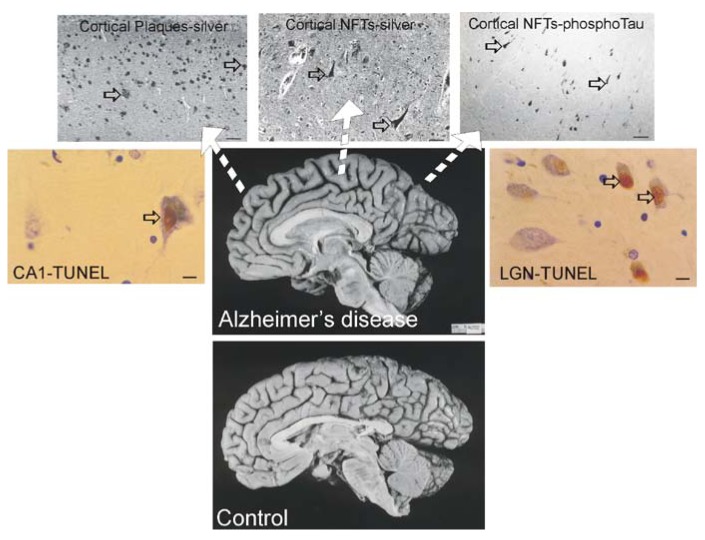
Brain atrophy and neurodegeneration in people with AD. Midsagittal views (center pictures) of the brains from an 85-year-old individual with AD and an 86-year-old normal control individual. The microscopic neuropathological hallmarks of AD are senile plaques (scale bar = 200 μm), neurofibrillary tangles (NFTs, scale bar = 50 μm.), and neuronal cell death determined by transferase-mediated biotin-dUTP nick-end labeling (TUNEL, brown nuclear staining, open arrows, scale bars = 10 μm) as seen in the hippocampus CA1 and in subcortical regions such as the thalamic lateral geniculate nucleus (LGN).

The dementia in AD is caused by severe atrophy of the cerebral cortex, as indicated by the widening of the sulci and narrowing of the gyri (Figure 3) while normal aged individuals have broad gyri and narrow sulci (Figure 3). Neurons in the neocortex, hippocampus, basal forebrain, and brainstem (e.g., dorsal raphe) are selectively vulnerable in AD [239,240,241,242,243]. The numerous lesions that are formed in the brains of AD patients are called senile plaques, containing abnormal extracellular deposits of Aβ amyloid protein (Figure 3, arrows), and NFTs which are abnormal intracellular aggregates of protein containing hyperphosphorylated tau (Figure 3, arrows).

It is useful to view the neurodegeneration in AD in the context of neural systems and stages [244]. Surprisingly, the classification of the neurodegeneration in AD is still not clear [245]. Dying neurons are found in cortical and subcortical regions in the AD brain (Figure 3). By TUNEL, an *in situ* DNA fragmentation/damage assay, subsets of neocortical, hippocampal, and thalamic neurons in the AD brain can be found with double-stranded DNA breaks, suggesting that these cells are in the process of dying (Figure 3). This neuronal death has been interpreted as apoptosis [246,247,248]; however, this DNA damage is not specifically indicative of apoptosis [17,24,249]. Other reports conclude that apoptosis does not have a major role in the neuronal degeneration of AD [250]. Experiments on changes in the levels of proteins in the Bcl-2 family in AD postmortem brain are difficult to interpret, with studies showing up-regulation of both anti-apoptotic and pro-apoptotic proteins or no changes [247,251,252]. AD brain degeneration might involve caspase-3 activation as determined by the imunohistochemical detection of cleaved caspase-3 [249,251,253] and *caspase* gene expression [254]. Cleaved caspase-3 [253], caspase-9 [255] and caspase-6 [256] have been found in NFT-bearing neurons in AD. However other studies have not shown evidence for the accumulation of cleaved caspase-3 in neurons in the AD brain [257], but changes seen in early and late AD may differ [253]. It is also noteworthy that immunodetection of cleaved caspase-3 is not always equivalent to caspase-3 activation as determined biochemically [258]. Furthermore, caspase-3 functions in processes other than cell death, including neuronal differentiation, migration, and plasticity [259,260]. 

Neuronal cell degeneration in AD occurs over a lengthy period. When considering the pathological classification of the primary neurodegeneration in AD it appears safe to conclude that based on morphology it is not classical apoptosis or necrosis. Autophagy could have a role in this neuronal cell death [249,261]. It might be more useful to consider neurofibrillary cell death separate from classical apoptosis and necrosis with some overlap in mechanisms according to the cell death matrix (Figure 2 and Figure 3). It could be important to know how neurons with neurofibrillary degeneration escape classical apoptosis and necrosis.

The mechanisms that cause the profound degeneration and loss of neurons in AD are not known, and existing information is incomplete. Abnormal processing or modification of APP and the cytoskeletal protein tau (a microtubule-associated protein) are involved in the pathogenesis (Figure 3) [262] resulting in amyloid (Aβ) deposits and neurofibrillary changes consisting of paired helical filaments, NFTs and dystrophic neurites (Figure 3) [262]. Cortical and hippocampal neuronal degeneration could be the consequence of a combination of several mechanisms including perturbations in protein metabolism, excitotoxicity, oxidative stress, mitochondrial perturbations, and inflammation. The possible specific mechanisms for neuronal degeneration in AD may involve dysfunction of NMDA receptors [264,265], dysregulation of Ca^2+^ and mitochondrial homeostasis [266,267], defects in synapses [268,269,270,271,272], abnormalities in the metabolism of APP and presenilin proteins, toxic actions of Aβ protein derived from APP [273,274], and cytoskeletal pathology [275,276]. 

There are possible disease links between intraneuronal Aβ and mitochondria suggesting an intracellular toxicity of Aβ [277,278,279]. Importantly, APP possesses a targeting sequence for mitochondria [277]. When over-expressed in cultured cells, APP interacts with mitochondrial import proteins, can arrest mitochondrial import, and can result in bioenergetic deficits [277]. In postmortem human brain samples, APP variants were found associated with mitochondria from the AD brain, but not mitochondria from control brain [278,280], and APP can interact with the translocase of the outer mitochondrial membrane (TOMM40) and TIMM23 [278]. The human AD autopsy brain shows evidence for mitochondrial impairments (for review see [267]). High mitochondrial APP levels mirror abnormalities in respiratory chain subunit levels and activity and enhanced ROS production [278]. Aβ can interact with the mitochondrial matrix protein Aβ-binding alcohol dehydrogenase in human AD brain and is believed to participate in mitochondrial dysfunction and oxidative stress [281]. A possible intraneuronal Aβ-mitochondria link was shown by EM in aged non-human primate neocortex [270]. 

In an APP transgenic (tg) mouse line (Tg2576) Aβ was also found to associate with mitochondria isolated from cerebral cortex [279]. It has also been reported that Aβ interacts with CyPD in mouse and human cerebral cortex mitochondria to potentiate synaptic stress [282]. Genetic deletion of CyPD (*ppif*) in human mutant APP tg mice (J-20 line) protects neurons from Aβ- and oxidative stress-induced cell death [282]. However, these abnormalities might not be related to the mPTP-driven cell death because these mice, and most other mouse models of AD, show scant or modest evidence for neurodegeneration resulting in neuronal cell death, despite tremendous brain burden of Aβ [283].

#### Most Tg Mouse Models of AD are not Useful to Study Neuronal Cell Death

Animal experiments should provide critical insight into to the mechanisms of neurodegeneration in AD. However, most human APP and/or presenilin tg mice show substantial Aβ deposits in hippocampus and cortex, but do not develop robust neuronal loss [284,285,286]. Analysis of a different tg mouse line (APP23) showed that Aβ deposition is accompanied by a modest loss of CA1 neurons but no cortical neuron loss, despite an Aβ burden similar to that seen in CA1 [287]. None of these studies have found the formation of NFTs. Cleaved caspase-3 was not found in mutant APP^Swedish^ tg mice [288], but sparse cleaved caspase-3-positive cells were found in mutant APP/presenilin mice [289]. Triple-tg mice harboring mutant presenilin, APP, and tau transgenes have intraneuronal accumulations of Aβ and phosphorylated tau, but neuronal loss was not reported [290]. However, in tg mice over-expressing Aβ protein, extracellular deposition Aβ and neuronal cell death is observed [291]. The neuronal cell death mechanism in these mice may involve p53 [291], consistent with cell culture findings that Aβ neurotoxicity involves p53 [292]. 

#### Cell Culture Models of Cortical and Hippocampal Neuron Cell Death and Interactions between APP, Aβ, Tau, and Caspases

It has long been thought by many researchers that Aβ is the primary cause of AD [272]. Extracellular application of Aβ can induce apoptosis [293] or necrosis [294] in cultured neurons. Extracellularly applied Aβ_25–53_ or Aβ_1–40_ induces mitochondrial dysfunction in primary cortical neurons [295], enhanced production of free radicals, intracellular Ca^2+^ destabilization, and DNA damage [296,297]. Studies of the specific intracellular signaling pathways that are activated by Aβ to trigger cell death are only now appearing in the literature. In studies of rodent neurons exposed to extracellular Aβ, the induction of apoptosis involves the Fas death receptor-Jun N-terminal kinase (JNK) pathway [298], the p75^NTR^-JNK pathway [299], and caspase-12 [300]. Neuronal cultures exposed to Aβ can be protected from neurotoxicity by caspase-8 inhibition and expression of dominant-negative Fas-associated protein with death domain (FADD), both components of the Fas death receptor pathway [301]. Some *in vitro* experiments have implicated p53 in neuron death trigged by extracellular Aβ [302,303], but other *in vitro* studies with Aβ-treated cortical neurons reveal a p53-independent mechanism for cell death involving the transcription factor E2F1 [304]. It is noteworthy that results of cell culture experiments using extracellular application of Aβ are likely to be dependent on Aβ concentration and oligomeric state. When human neuron primary cultures are treated with Aβ at concentrations closer to physiological levels for up to 3 days, evidence for apoptosis is scarce; however, Bcl-2 levels are down-regulated and Bax levels are up-regulated [305]. Thus, extracellular Aβ at physiological concentrations might render neurons more sensitive to cell stress rather than kill them outright. Intracellular Aβ_1-42_ exposure (as little as 1 picomolar) is, in contrast, toxic to human cortical neurons, and this toxicity requires *de novo* protein synthesis, Bax, p53, and caspases, suggesting cell death by some form of apoptosis [292].

Experiments have been performed to link APP and presenilins to cell death. Over-expression and intracellular accumulation of APP activates caspase-3 [306]. APP is a target of caspase-3 [306] and caspase-6 [307], and APP cleavage by caspase-3 or caspase-6 may promote Aβ formation [307,308]. Thus, increased production of Aβ may be a consequence of neuronal apoptosis. Recent work has shown that shedded N-terminal fragments of APP are surface ligands that bind death receptor 6 (DR6) to trigger axon pruning through caspase-6 and neuron death through caspase-3 [309]. Neurotrophin deprivation engages the shedding of APP to activate DR6 [309]. Presenilin proteins can influence mitochondrial regulation of apoptosis, such as Bax activation and cytochrome *c* release, through interactions with Bcl-X_L_ [310], and they are also substrates for caspase-3 [311]. However, over-expression of human wild-type or mutant presenilin-1 or presenilin-2 does not enhance apoptosis in neurons [312,313]. Other work indicates that the presenilin-1 mutation sensitizes neurons to DNA damage-induced apoptosis [314]. More cell culture work needs to be done on the basic mechanisms of human neuron degeneration and on APP and Aβ neurotoxicity mechanisms under basal conditions and in the presence of familial AD-related and tau gene mutations.

### Parkinson’s Disease (PD)

PD is a chronically progressive, age-related, fatal neurological disease in humans described first by James Parkinson in 1817. Estimates indicate that 4 to 6 million people have been diagnosed with PD. It affects about 2% of the population at some time in life. The greatest prevalence occurs in the USA, with between 100 and 250 cases per 100,000 [315], placing PD as the 2^nd^ most common neurodegenerative disease with an adult onset (after AD). Progressive resting tremor (4–7 Hz), rigidity, bradykinesia/akinesia, gait disturbance, and postural instability characterize PD clinically [316]. The disease progression is also associated with mood disturbances, dementia, sleep disturbances, and autonomic dysfunction [316]. There are currently no cures for PD. Medications and neurosurgery can relieve some of the symptoms.

**Figure 4:**
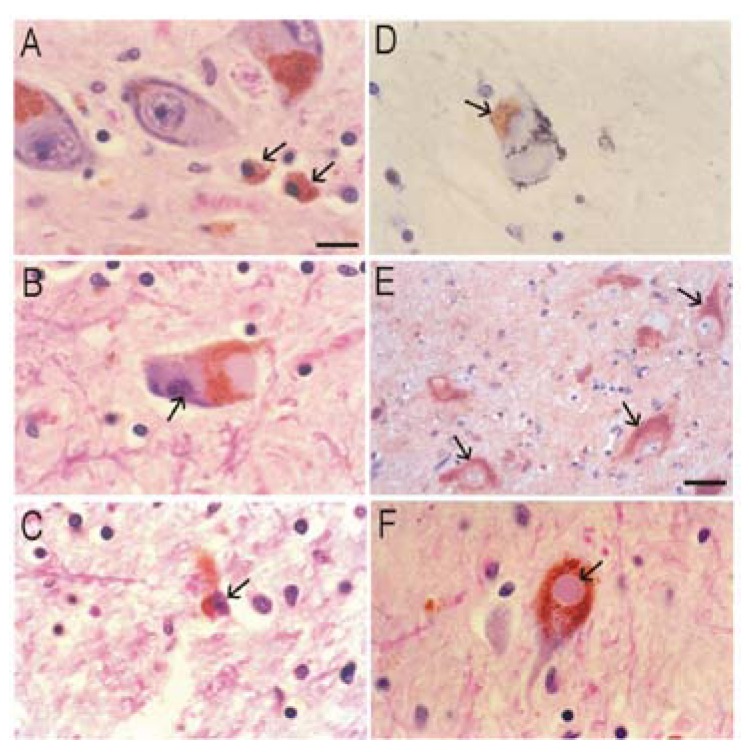
Degeneration of SNc neurons in human PD. **A-C. **Hematoxylin-eosin (H&E) staining shows that SNc neuron degeneration is characterized by chromatolysis (A), nuclear condensation (B), and severe soma attrition (C). **D.** Pigmented SNc neurons accumulate DNA double-strand breaks (arrow, brown staining). **E.** SNc neurons accumulate cleaved caspase-3 immunoreactivity (arrows, brown staining). **F.** SNc neurons can form Lewy bodies (arrow). Scale bars: A, 20 μm (same for B-D, F); E, 45 μm.

A major neuropathological feature of PD is the degeneration and elimination of dopamine neurons in substantia nigra pars compacta (SNc) and in other brainstem regions which causes the movement disorder (Figure 4) [317]. The movement disorder in PD is thought to arise from reduced dopaminergic innervation of the striatum resulting from the loss of SNc neurons [317]. The effect of reduced dopaminergic input is over-activity of striatal neurons that project to and inhibit neurons in external globus pallidus (GPe), thus reducing the normal GPe inhibition of excitatory subthalamic neurons [2]. In addition, due to actions of dopamine on different dopamine receptor subtypes, there is also loss of normal dopaminergic excitation of striatal neurons that innervate internal GP (GPi) and SN reticularis, causing increased γ-aminobutyric acidergic inhibition of thalamic nuclei that are needed to drive cortical activation [317]. PD can thus be explained functionally by over-activity of the subthalamic nucleus and GPi [317]. PD should however be regarded as a multi-regional, multi-system neurodegenerative disorder in which the pathology appears in a regionally specific sequence, beginning in the dorsal motor nucleus of the vagus and olfactory bulbs and anterior nucleus followed by the locus coeruleus and then the SNc, at which time (when ~ 50% of SNc neurons are lost) a clinical diagnosis of PD becomes possible [318].

The degeneration of pigmented SNc neurons is characterized by chromatolysis (Figure 4A), nuclear condensation (Figure 4B), and severe soma attrition (Figure 4C). The neuronal chromatolysis (Figure 4A) is indicated by the eccentrically placed nucleus, pale cytoplasm, and peripheral margination of the Nissl substance. Glial/macrophage-like cells (Figure 4A, arrows) are laden with phagocytosed cellular debris. The nucleus of SNc neurons undergoes considerable condensation (Figure 4B, arrow) while the Nissl substance dissipates, but before appreciable somal shrinkage. The cell body of SNc neurons then becomes attritional (Figure 4C, arrow), resulting in residual neurons that are ~10–20% their normal size. Cells can be identified as atrophic neurons, rather than a debris-laden macrophage, because of the presence of a condensed nucleus with a single prominent nucleolus (Figure 4C). This degeneration pattern could be indicative of autophagy. The nuclear condensation stage of pigmented SNc neuron degeneration is characterized by the appearance of DNA double-strand breaks (Figure 4D), and in the chromatolytic stage SNc neurons accumulate cleaved caspase-3 immunoreactivity (Figure 4E) prior to their undergoing nuclear condensation and somal attrition. Another neuropathological feature of PD is the formation of eosinophilic proteinaceous intra-neuronal or intra-glial inclusions (Figure 4F, arrow), known as Lewy bodies (LBs), first described by Frederich Lewy in 1912. LBs are comprised of a dense core of filamentous material enshrouded by filaments 10–20 nm in diameter and are usually positive for ubiquitin and α-synuclein (α-Syn) [319]. It is not clear whether LBs are related causally to the disease process.

The molecular pathogenesis of PD is still not understood. At least 2 forms of PD exist: idiopathic (sporadic) and heritable (familial) [320]. The majority of PD cases are sporadic with no known genetic component. Epidemiological studies reveal several risk factors for developing idiopathic PD in addition to aging. Pesticides have now been linked convincingly to the development of PD [321]. Herbicides, well water (contaminated with pesticides), and industrial chemicals are possible neurotoxic agents related to the development of PD [320].

**Table 3:** Mutant Genes Linked to Familial PD.

Locus	Inheritance	Gene	Protein Name/ Function
PARK1/4q21	autosomal dominant	α-syn	α-Syn/presynaptic maintenance?
PARK2/6q25.2-27	autosomal recessive	parkin	Parkin/ubiquitin E3 ligase
PARK3/2p13	autosomal dominant	?	?
PARK4/4p15	autosomal dominant	α-syn	α-Syn/presynaptic maintenance?
PARK5/4p14	autosomal dominant	UCHL1	UCHL1/polyubiquitin hydrolase
PARK6/1p36	autosomal recessive	PINK1	PTEN-induced putative kinase-1/mitochondrial protein kinase
PARK7/1p36.33-36-12	autosomal recessive	DJ-1	DJ-1/mitochondrial antioxidant, chaperone
PARK8/12q12	autosomal dominant	LRRK2	Dardarin/multifunctional kinase/GTPase
PARK9/1p36	autosomal recessive	ATP13A2	Lysosomal type 5 P-ATPase
PARK10/1p32		?	?
PARK11/2q36-37	autosomal dominant	GIGYF2?	Grb10-interacting GYP protein 2, modulates tyrosine kinase receptor signaling, including IGF-1
PARK12/Xq21-q25	X-linked	?	?
PARK13/2p12	autosomal recessive susceptibility factor	Omi/HtrA2	Omi/HtrA2, mitochondrial serine peptidase, inhibitor of IAPs
PARK14/22q13.1	autosomal recessive	PLA2G6	Phospholipase A2 group VI
PARK15/22q12-q13	autosomal recessive	FBXO7	F-box protein 7

#### Mutant Genes that Cause Some Forms of PD

About 5–10% of PD patients have familial patterns of inheritance [320]. Several genes have been identified in Mendelian forms of PD. Gene mutations with autosomal dominant or autosomal recessive inheritance patterns have been identified in familial forms of PD (Table 3). PD-linked mutations occur in the genes encoding α-Syn, Parkin, ubiquitin carboxy-terminal hydrolyase-L1 (UCH-L1), phosphatase and tensin homolog (PTEN)-induced putative kinase-1 (PINK1), DJ-1, and leucine-rich repeat kinase-2 (LRRK2). In rare autosomal dominant inherited forms of PD, missense mutations in the *α-syn* gene (*PARK1*) result in amino acid substitutions Ala-53→Thr, Ala-30→Pro, Glu-46→Lys; in addition, duplication and triplication mutations in the *α-syn* gene (*PARK4*) have been found [322,323,324]. A missense mutation in the *UCH-L1* gene (*PARK5*), resulting in the amino acid substitution Ile-93→Met, can also cause very rare autosomal dominant PD [325]. Loss-of-function mutations due to large deletions and truncations and also missense or nonsense mutations in *parkin* (*PARK2*), *PINK1* (*PARK 6*), and *DJ-1* (*PARK7*) are the cause of autosomal-recessive inheritance of PD [326,327,328,329]. Several missense mutations in the *LRRK2* gene (*PARK8*) have been found, resulting in amino acid substitutions Tyr-1654→Cys, Arg-1396→Gly, Tyr-1699→Cys, Arg-1441→Cys, Ile-1122→Val, Ile-2020→Thr, that cause more commonly occurring autosomal dominant PD and possibly ‘sporadic’ PD [330,331]. *PARK9* has been ascribed to a deletion mutation (cytosine at nucleotide position 3057) or guanine-to-adenine transition at a splice site of exon 13 in the *ATP13A2* gene that encodes a predominantly neuronal P-type lysosomal ATPase [332]. Potential lysosomal dysfunction related to ATP13A2 mutant proteins might tie into PD etiology through abnormalities in autophagy. Mutations of genes at other PD loci are more controversial (Table 3).

#### α-Syn

α-Syn is a relatively small (140 amino acids) very abundant protein (~1% of total protein) found in cells throughout the nervous system and is particularly enriched in neuronal axon terminals [333,334,335]. The growing evidence shows a role for α-Syn in neurotransmitter release. Mice without α-Syn have no overt phenotype [337], but neurons deficient in α-Syn have a reduction in the reserve pool of synaptic vesicles needed for responses to tetanic stimulation and show defective mobilization of dopamine and glutamate [335,338]. Without α-Syn neurons have impaired long-lasting enhancement of evoked and miniature neurotransmitter release [339]. α-Syn is highly mobile and rapidly dissociates from synaptic vesicle membranes after fusion in response to neuronal activity [340]. It appears that α-Syn acts as a molecular chaperone to assist cysteine-string protein-α (alias for DNAJC5) in the folding and refolding of SNARE synaptic proteins [341].

α-Syn is a soluble monomeric protein that can associate with mitochondrial membranes [342]. α-Syn can be induced to polymerize into insoluble fibrils due to a conformational change from an α-helical coil to a β-pleated sheet [343]. α-Syn is a major structural component of LBs (Figure 4F), forming the ~10-nm fibrils, but in most neurodegenerative diseases, LBs are associated with accumulation of wild-type, not mutant, α-Syn [319]. α-Syn mutations cause increased levels of protofibrils, possibly being the more toxic form of the protein [344]. α-Syn protofibrils might also be toxic by making membranes of cells more porous [345]. Over-expression of human wild-type or mutant α-Syn in cultured cells elevates the generation of intracellular ROS [346,347] and causes mitochondrial deficits [346]; moreover, expression of mutant α-Syn increases cytotoxicity to dopamine oxidation products [348]. Aggregation of wild-type and mutated α-Syn is associated with enhanced cell death in cultured cells [349]. Nitration of α-Syn, signifying the presence of potent reactive nitrogen species such as ONOO^-^ or its free radical derivative nitrogen dioxide (NO_2_), is a major signature of human PD and other synuclinopathies and might be critical to the aggregation process [350,351].

#### UCH-L1 and Parkin

UCH-L1 is a very abundant protein (~1–2% total soluble protein in brain) that functions in the formation and recycling of ubiquitin monomers for the ubiquitin-proteasome pathway [352]. This pathway is important for intracellular protein turnover and degradation and functions generally in quality control of proteins in cells to eliminate misfolded, mutated, and damaged proteins [353]. Ubiquitin is an abundant small (~8.5 kDa) protein that is attached covalently to lysine aliphatic chains in proteins to mark them for degradation carried out by the 26S proteasome. UCH-L1 hydrolyses the C-terminus of fusion proteins containing polyubiquitin molecules and ribosomal protein, thus generating ubiquitin monomers. *In vitro*, PD-linked mutant UCH-L1 has reduced enzyme activity [354], and inhibition of UCH-L1 is associated with production of α-Syn aggregates [355], indicating that α-Syn is degraded by the proteasome. 

The ubiquitination of proteins is catalyzed by the activities of three enzymes called ubiquitin-activing enzyme (E1), ubiquitin-conjugating enzyme (E2), and ubiquitin ligase (E3) [353]. *PARK2*, encoding Parkin, a ubiquitin E3 ligase, causes juvenile-onset recessive PD (before 40 years of age) [328] with relatively confined neuronal loss in the SNc and locus coeruleus, but with an absence of LBs. Several substrates of Parkin have been identified, including α-Syn, synphilin-1 and other synaptic proteins [356]. Mutations in the *parkin* gene result in a loss-of-function of E3, thus possibly causing some substrates of Parkin to accumulate and aggregate within cells. One *parkin* mutation found in a Turkish patient (Gln-311→X), replacing a glutamine residue at position 311 with a stop codon), causes a C-terminal truncation of 155 amino acids of Parkin [357].

#### PINK1

The *PARK6* locus contains the *PINK1* gene [327,329]. *PARK6* kindred have juvenile-onset PD and truncation mutations or missense mutations in the *PINK1* gene resulting in truncation or single amino acid substitutions in the PINK1 protein (His-271→Gln; Gly-309→Ala; Leu-347→Pro; Glu-417→Gly) as well as mutations that are nonsense (Arg-246→X, where X is any other amino acid; Trp-437→X) or compound nonsense (Gln-309→X/Arg-492→X). Clues about the intracellular localization and normal functions of PINK1 are emerging. It is a 581 amino acid protein (~63 kDa) and contains a domain highly homologous to the serine/threonine protein kinases of the calcium/calmodulin family and a mitochondrial targeting motif [329]. Thus, PINK1 is a mitochondrial kinase. It is processed at the N-terminus in a manner consistent with mitochondrial import, but the mature protein is also present in the cytosol [358]. Both human wild-type and mutant PINK1 localize to mitochondria [359]. Interestingly, most of the reported mutations are in the putative kinase domain. PINK1 is expressed in many adult human tissues [360]. In adult rodents PINK1 is expressed throughout the brain [361]. 

PINK1 appears to function in mitochondrial trafficking by forming a multiprotein complex with the GTPase Miro and the adaptor protein Milton [362]. Speculation has PINK1 protecting human dopaminergic neuroblastoma cells (SH-SY5Y) against mitochondrial malfunction under conditions of cell stress [363]. In rat neuroblastoma cells, mutant PINK1 can induce abnormalities in mitochondrial Ca^2+^ influx and aggravate the cytopathology caused by mutant α-Syn in a mechanism that involves the mPTP [364]. It is unclear how PINK1 mutations cause the selective death of SNc neurons in PD.

#### DJ-1

The *PARK7* locus contains the *DJ-1* gene [326]. *PARK7* kindred can have homozygous deletion of a large region within the *DJ-1* gene causing complete loss of DJ-1 expression or homozygous missense mutations in the *DJ-1* gene resulting in single amino acid substitutions in the DJ-1 molecule (Met-26→Ile; Glu-64→Asp; Leu-166→Pro) [326]. DJ-1 is a small (189-amino acid, ~20–25 kDa) protein with multiple apparent functions involving cellular transformation, male fertility, control of protein-RNA interaction, and oxidative stress response. The protein exists *in vivo* as a dimer [326], and information on its specific functions is growing. DJ-1 is expressed throughout the mouse nervous system [366], and it might act as a neuroprotective intracellular redox sensor that can localize to the cytoplasmic side of mitochondria [367]. The localization of DJ-1 to mitochondria is associated with protective actions against some mitochondrial poisons [367]. Some DJ-1 mutants have abnormalities in dimer formation ability and decreased stability [368,369]. It remains to be identified how DJ-1 mutations cause the selective death of SNc neurons in PD.

#### LRRK2

The *PARK8* locus contains the *LRRK2* gene [330,331]. Mutations in this gene are to date the most common in both familial and “sporadic” PD. The LRRK2 protein is a large multidomain protein (2527 amino acids, 286 kDa), also called dardarin (derived from the Basque word dardara, meaning tremor), that is expressed throughout the body. LRRK2 contains leucine-rich repeat domains, a Ras/small GTPase domain, a non-receptor tyrosine kinase-like domain, and a WD40 domain (~40 amino acid motifs often terminating in a Trp-Asp dipeptide), consistent with the architecture of multifunctional Ras/GTPases of the Ras of complex (ROC) family. The presence of leucine-rich and WD40 domains suggests that LRRK2 is capable of multiple protein-protein interactions. The GTPase activity indicates that LRRK2 functions as a molecular switch, possibly involved in cytoskeleton organization and vesicle trafficking. The kinase domain possibly belongs to the mitogen-activated protein kinase kinase kinase (MAPKKK or MEKK) family of kinases. 

Currently it is not evident how *LRRK2* gene mutations relate to the selective death of neurons that causes PD. Studies of rodent brain show little or no expression of LRRK2 in SNc neurons [370,371]; however, expression of LRRK2 is high in dopamine-innervated regions [370]. Recent work shows that LRRK2 can influence mitochondrial- and death receptor-mediated cell death in cultured cells [372,373]. These findings might be hints that the target of SNc neurons (*i.e.* the striatum) and SNc neuron target-deprivation are important to the understanding of LRRK2-related pathogenic mechanisms of PD. 

#### Neuronal Cell Death in Human PD

Many groups of neurons are affected in PD [318], but the loss of dopaminergic neurons in the SNc is particularly disease manifesting. The mechanisms by which nigral neurons degenerate in human PD are not well understood and require further study for the development of effective cures. The pathology seen in autopsy tissue of the SNc in human PD shows atrophy, degeneration, and loss of large, multipolar melanin-containing neurons (Figure 4). Some studies report that apoptotic PCD contributes to the neurodegeneration in human PD based on the presence of cleaved caspase-3 (Figure 4E) [374], but other studies caution against such claims [375,376]. Nigral neurons in human PD do not degenerate with the morphology consistent with the process of classical apoptosis (Figure 4A-C); however, cell death exists as a morphological continuum [4,28,29] and non-apoptotic forms of PCD exist [27,46,47,48]. The pathological process in PD might involve autophagy [377,378]. Studies using *in situ* DNA-end labeling methods to detect dying cells with DNA fragmentation in the SNc of humans with PD are conflicting. Some studies report no labeling [375], but other experiments have found nuclear DNA fragmentation [374,379]. Using TUNEL, subsets of large, melanin-containing, atrophic neurons with DNA double-strand breaks can be found (Figure 4D). Other neurons without melanin are TUNEL-positive, including glial and macrophage-like cells. 

Confusion about neuronal cell death mechanisms in the PD brain originates from how PCD is defined, the use of detection systems that do not distinguish between types of DNA-strand breaks associated with apoptosis or necrosis, arbitrary morphological interpretations of chromatin condensation, and uncertainty about the meaning of caspase immunoreactivities in cells [17,24,375]. Studies are warranted that utilize high-resolution techniques, such as laser capture microdissection, with unambiguous selectivity for specific neurons and proteomic approaches [380,381] to reveal quantitatively mechanisms of cell death, such as the activation of apoptotic, autophagic and programmed necrotic pathways, directly in SNc neurons in human autopsy tissue and optimally prepared animal model tissue. 

#### PD α-Syn Tg Mice Develop Neuronal Mitochondrial Degeneration and Cell Death

Information gleaned from molecular genetic studies of human genes linked to familial PD drives experimental work on the generation of animal and cell models of PD. *Parkin* null mice appear to have a normal lifespan, do not develop any major neurological abnormalities, and have no loss of midbrain dopaminergic neurons and no formation of inclusions [382,383]. However, *parkin^-/-^* mice exhibit some evidence of dopaminergic presynaptic dysfunction in striatum and possible deficits in behavioral tests indicative of nigrostriatal dysfunction [382], although this finding has not been confirmed in another mouse line [383]. *Parkin^-/-^* mice have decreases in proteins involved in mitochondrial oxidative phosphorylation and oxidative stress in ventral midbrain and exhibit reduced mitochondrial respiration in striatum, but they have no mitochondrial ultrastructural abnormalities [384]. In contrast, tg mice expressing the Parkin Q311X truncation mutation develop a progressive hypokinetic disorder, degeneration of SNc neurons, and loss of striatal dopamine [385]. Thus, Parkin could be important for maintenance of mitochondrial function or mitochondrial turnover through autophagy and synaptic integrity distally within the SNc neuron target region. 

Mice with null mutations in *DJ-1* also have a normal lifespan and do not develop an overt phenotype or loss of dopaminergic neurons, but behavioral tests reveal age-dependent motor deficits and neurochemical assessment shows altered striatal dopamine content [386]. *DJ-1* null mice also show altered D2 dopamine receptor-mediated function [387]. 

Several tg mouse lines have been made using different promoters to drive expression of human full-length wild-type or mutant α-Syn [349,388,389,390,391,392,393]. Of these mouse lines, tg mice expressing human A53T mutant α-Syn have a shortened lifespan and develop a severe movement disorder and synucleinopathy [349,390,393]. It is noteworthy that there have been no reports of robust dopamine SNc neuron degeneration in full-length α-Syn tg mice. However, tg mice expressing a truncation mutant of human α-Syn show a development-related loss of SNc neurons [394]. Cell death mechanisms or thresholds for cell death activation in human and mouse brain dopamine neurons might differ. 

Despite the absence of prominent changes in the SNc, tg α-Syn mice do develop robust cell death and neuronal loss in other regions of brain and in spinal cord [395]. These tg mice express high levels of human wild-type or mutant (A53T and A30P) α-Syn under the control of the mouse prion protein promoter [390]. Mice expressing A53T α-Syn (lines G2-3 and H5), but not mice expressing wild-type (line I2-2) or A30P (line O2) α-Syn, develop adult-onset progressive motor deficits, including reduced spontaneous activity with bradykinesia, mild ataxia and dystonia, at ~10–15 months of age followed by rapidly progressive paralysis and death [390]. A53T mice develop intraneuronal inclusions, mitochondrial degeneration, and cell death in neocortex, brainstem, and spinal cord [395]. Brainstem neurons and spinal motor neurons display a prominent chromatolysis reaction and axonal spheroids, typical of that seen after axonal injury [396]. A53T mice form LB-like inclusions in neocortical and spinal motor neurons and have progressive, profound loss (~75%) of motor neurons that causes their paralysis [341,395]. 

Mitochondrial pathology in A53T mice involving mitochondrial DNA damage is seen frequently in the absence of nuclear DNA damage in large brainstem neurons and spinal motor neurons [395]. Subsets of mitochondria in brainstem and spinal cord cells in A53T mice appear dysmorphic, becoming shrunken, swollen, or vacuolated [395]. Human α-Syn is found bound to some mitochondria in degenerating neurons in A53T mice [395]. Some abnormal intracellular inclusions in these cells are degenerating mitochondria. A mitochondrial defect in A53T mice is further indicated by biochemical evidence revealing loss of complex IV activity [395]. 

The mechanisms for this mitochondrial DNA damage are possibly related to oxidative stress. The presence of oxidative stress in mutant α-Syn tg mice is shown by evidence that mitochondrial associated metabolic proteins are oxidized in A30P mice [397]. α-Syn can generate H_2_O_2_ [398] and ^•^OH [399] *in vitro* upon incubation with Fe(II). There is evidence for ONOO^-^-mediated oxidative/nitrative stress in A53T mouse motor neurons, as indicated by the presence of nitrated human synuclein [395]. Nitrated synuclein forms inclusions in motor neurons consistent with *in vitro* data showing that ONOO^-^ promotes the formation of stable α-Syn oligomers [400,401]. The evidence for mitochondrial DNA damage is consistent with the presence of ONOO^-^ or its derivatives near mitochondria, because ONOO^- ^or products of ONOO^-^ are directly genotoxic by causing single- and double-strand breaks in DNA [13]. Moreover, the loss of complex IV enzyme activity without a change in protein level [395] might be explained by inactivation of this mitochondrial enzyme by nitration. Overall, ONOO^-^ mediated damage in mitochondria could be a key pathological mechanism leading to motor neuron degeneration in A53T mice. 

The reasons for the vulnerability of mouse motor neurons to human A53T mutant α-Syn are not clear. These mice express high levels of mRNA and protein for human α-Syn in the forebrain, diencephalon, and midbrain [390], but these regions are much less vulnerable than spinal cord. A53T α-Syn causes axonopathy [349,393], thus motor neuron vulnerability could be related to their long myelinated axons and interactions with oligodendrocytes and Schwann cells for myelin support. Motor neuron vulnerability could also be related to their unusual expression of inducible NOS (iNOS) in mitochondria [381,402]. Moreover, distal axonopathy and muscle disease may have roles in the pathogenesis in A53T mice [395]. Prominent skeletal muscle denervation occurs in α-Syn tg mice [393,395]. This work is intriguing because the original goal was to develop a tg mouse model of PD, but the result is a mouse model of ALS. Thus, the mutant α-Syn A53T tg mouse is a new model to study mechanisms of motor neuron degeneration and could provide insight into the selective vulnerability of motor neurons in age-related disorders and the possible roles of α-Syn in synaptic maintenance and diseases of long-axon neurons [395]. 

### ALS

**Figure 5:**
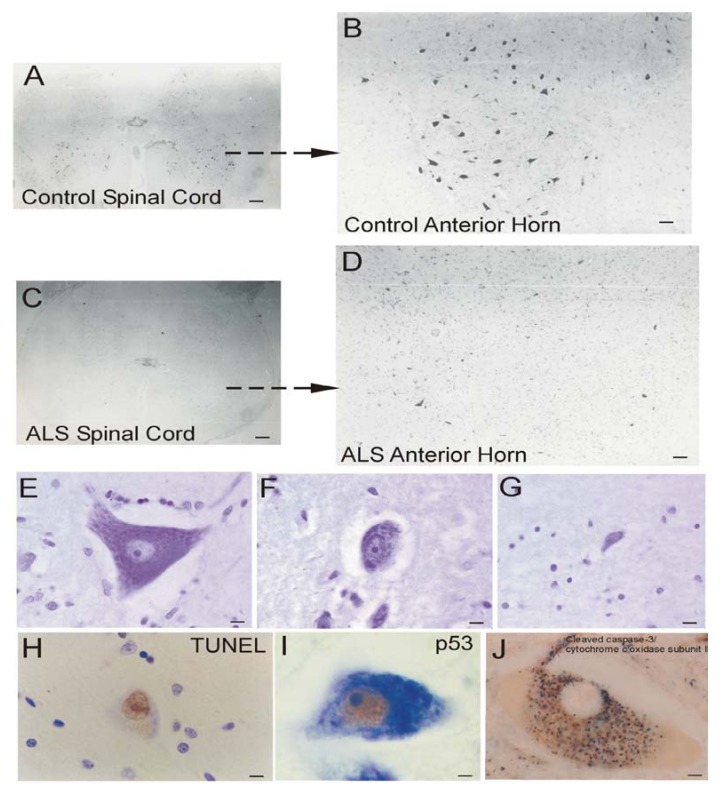
Motor neurons in spinal cord degenerate in people with ALS. **A, B**. In normal individuals, the spinal cord contains many large, multipolar motor neurons (dark cells). **C, D**. In ALS, the anterior horn is depleted of large neurons (dark cells) and remaining neurons are atrophic. **E-G. **Nissl staining shows motor neurons that appear normal (E), attritional (F), and residual (G). **H.** ALS motor neurons accumulate DNA double-strand breaks in the nucleus (brown labeling). **I.** p53 (brown labeling) accumulates in the nucleus of motor neurons. **J. **ALS motor neurons are immunopositive for cleaved caspase-3 (black-dark green labeling), and discrete mitochondria (brown-orange labeling, detected with antibody to cytochrome *c* oxidase subunit I) accumulate around the nucleus (pale circle). Scale bars: A, 500 μm (same for C); B, 76 μm (same for D); E, 6 μm (same for F,G); H, 12 μm (same for I,J).

ALS is a progressive and severely disabling neurological disease in humans characterized by initial muscle weakness, and then muscle atrophy, spasticity, and eventual paralysis and death typically 3 to 5 years after symptoms begin [403]. The cause of the spasticity, paralysis and death is progressive degeneration and elimination of upper motor neurons in cerebral cortex and lower motor neurons in brainstem and spinal cord (Figure 5) [403,404]. Degeneration and loss of spinal and neocortical interneurons has also been found in human ALS [405,406]. More than 5000 people in the USA are diagnosed with ALS each year (ALS Association, http://www.alsa.org), and, in parts of the United Kingdom, three people die every day from some form of motor neuron disease (http://www.mndassociation.org). Other than life support management, no effective treatments exist for ALS [225].

It is still not understood why specific neuronal populations are selectively vulnerable in ALS, such as certain somatic motor neurons and interneurons [404,405,406]. The molecular pathogenesis of ALS is understood poorly, contributing to the lack of appropriate target identification and effective mechanism-based therapies to treat even the symptoms of this disease. Two forms of ALS exist: idiopathic (sporadic) and heritable (familial). The majority of ALS cases are sporadic with few known genetic contributions, except for missense mutations in TAR-DNA binding protein [407]. Aging is a strong risk factor for ALS because the average age of onset is 55 (ALS Association, www.alsa.org). Familial forms of ALS have autosomal dominant or autosomal recessive inheritance patterns and make up ~10% or less of all ALS cases. ALS-linked mutations occur in the genes (Table 4) encoding SOD1 (*ALS1*), Alsin (*ALS2*), senataxin (*ALS4*), fused in sarcoma (FUS, *ALS6*), vesicle associated membrane protein (VAMP/synaptobrevin)-associated protein B (VAPB, *ALS8*), p150 dynactin (DCTN1), and TAR-DNA binding protein (TADBP or TDP43) [5,408]. Most recently, variations in the phosphoinositide phosphatase *FIG4* gene cause *ALS11* [409]. Several other genes are believed to be susceptibility factors for ALS (Table 4).

**Table 4:** Mutant/Polymorphic Genes Linked to Familial ALS.

Locus	Inheritance	Gene	Protein Name/ Function
ALS1/21q22	autosomal dominant (adult onset)	SOD1	Cu/Zn superoxide dismutase/ dismutation of superoxide
ALS2/2q33.2	autosomal recessive (juvenile onset primary lateral sclerosis)	Alsin	Alsin/guanine exchange factor for RAB5A and Rac1
ALS4/9q34	autosomal dominant (adult onset)	SETX	Senataxin/helicase, RNA processing
ALS6/16q12	autosomal recessive (adult onset)	FUS	Fused in sarcoma, component of heterogeneous nuclear ribonuclear protein complex; RNA/DNA binding protein
ALS8/20q13.33	autosomal dominant	VAPB	VAMP-associated protein B/part of SNARE complex
2q13	autosomal dominant (adult onset, atypical ALS)	DCTN1	Dynactin p150^glued^/axonal transport, link between dynein and microtubule network
ALS10/1p36.22	autosomal dominant	TARDBP	TAR DNA binding protein, DNA and RNA binding protein, regulates RNA splicing
ALS11/6q21	autosomal recessive	FIG4	FIG4 homolog, SAC1 lipid phosphatase domain containing protein; regulates phosphotidylinositol turnover
14q11.1-q11.2	susceptibility factor	ANG	Angiogenin; angiogenesis; stimulates production of rRNA
22q12.2	susceptibility factor	NEFH	Neurofilament, heavy polypeptide; neurofilament subunit
12q12-q13	susceptibility factor	PRPH	Peripherin; intermediate filament formation
5q13	susceptibility factor	SMN	Survival motor neuron; RNA processing
7q36.6	susceptibility factor?	DPP6	Dipeptidyl-peptidase 6; S9B serine protease, binds voltage-gated potassium channels

#### Mitochondrial Dysfunction in Human ALS

Mitochondrial abnormalities have been found in human ALS. Structural abnormalities in mitochondria are seen by electron microscopy in skeletal muscle, liver, spinal motor neurons and motor cortex of ALS patients [410,411]. A mutation in cytochrome *c* oxidase subunit I was found in a patient with a motor neurons disease phenotype [412]. Another patient with motor neuron disease had a mutation in a mitochondrial tRNA gene [413]. One type of mitochondrial DNA (mtDNA) mutation, called the common mtDNA deletion (mtDNA4977), is found non-uniformly within different human brain areas; the highest levels are detected in the striatum and SN [414,415]. However, no significant accumulation of the 5 kb common deletion in mtDNA has been found by single-cell analysis of motor neurons from sporadic ALS cases [416]. Some ALS patients with defects in skeletal muscle mitochondrial oxidative phosphorylation have a novel *SOD1* gene mutation [417]. More work on human ALS and animal/cell models needs to be done to determine conclusively if mitochondrial abnormalities participate in disease-causing mechanisms of human ALS.

Intracellular Ca^2+^ abnormalities and excitotoxicity are suspected links to mitochondrial dysfunction and oxidative stress in ALS. Mitochondria function in the regulation of intracellular Ca^2+^ levels [7,418]. Skeletal muscle biopsies of patients with sporadic ALS show ultrastructural changes indicative of elevated Ca^2+^ in motor neuron terminals, with some mitochondria showing an augmented Ca^2+^ signal [419]. Utilizing specific transport systems, mitochondria can pump Ca^2+^ from the cytosol into the matrix by the Ca^2+^ uniporter and eject Ca^2+^ from the matrix via the Na^+^/Ca^2+^ exchanger [7] and more catastrophically through the mPTP [50]. Under conditions of elevated cytoplasmic Ca^2+^, whenever the local free Ca^2+^ concentration rises above a set-point of ~0.5 μM, mitochondria avidly accumulate Ca^2+^ to a fixed capacity [7]. The electrical gradient across the IMM, the ΔΨ_m_, established by electron transport chain activity (Figure 2), provides the driving force for the accumulation of Ca^2+^ into the mitochondrial matrix [418]. Cytosolic Ca^2+^ concentrations above set-point levels are believed to be achieved during tetanic stimulation and by activation of glutamate receptors on the plasma membrane [7]. In pathological settings of excitotoxicity, resulting from excessive overstimulation of glutamate receptors [190,191], Ca^2+^ overload in neurons is significant and causes cell death [420]. When mitochondria become overloaded with Ca^2+^, they undergo mPT resulting in osmotic swelling and rupture of the OMM (Figure 1). Mitochondria within synapses appear to be more susceptible than non-synaptic mitochondria to Ca^2+^ overload [421].

Excitotoxicity has been implicated in the pathogenesis of ALS for a long time [422] and is another possible mechanism of motor neuron damage in ALS [420]. While many drugs targeting excitotoxicity as a mechanism have failed in ALS clinical trials, the anti-excitotoxic drug riluzole is currently approved by the Food and Drug Administration for ALS treatment. Many sporadic ALS patients have reduced levels of synaptosomal high-affinity glutamate uptake [422] and astroglial glutamate transporter EAAT2 (excitatory amino acid transporter 2 or GLT1) in motor cortex and spinal cord [423]. Reductions in levels of activity of EAAT2 in spinal cord could increase the extracellular concentrations of glutamate at synapses on motor neurons. Motor neurons might be particularly sensitive to glutamate excitotoxicity because they have a low proportion of GluR2-edited or under-edited AMPA subtype glutamate receptor on their surfaces, predisposing these cells to risk of excess Ca^2+^ entry and mitochondrial perturbations (Figure 1) [424,425]. Cell culture studies show that excess glutamate receptor activation in neurons can cause increased intracellular Ca^2+^, mitochondrial ROS production, bioenergetic failure, and mitochondrial trafficking abnormalities [426]. Ca^2+^-induced generation of ROS in brain mitochondria is mediated by mPT (Figure 1) [427]. Motor neurons are particularly affected by inhibition of mitochondrial metabolism which can cause elevated cytosolic Ca^2+^ levels and increased excitability [428].

Markers of oxidative stress and ROS damage are elevated in ALS tissues [429]. In human sporadic and familial ALS, protein carbonyls are elevated in motor cortex [430]. Tyrosine nitration (Figure 1) is elevated in human ALS nervous tissues [431,432,433]. Studies of respiratory chain enzyme activities are discrepant. Studies have shown increases in complex I, II, and III activities in vulnerable and non-vulnerable brain regions in patients with familial ALS-mutant SOD1 [434], but other studies show decreased complex IV activity in spinal cord ventral horn [435] and skeletal muscle [436] of sporadic ALS cases. In sporadic ALS skeletal muscle, reductions in activity of respiratory chain complexes with subunits encoded by the mitochondrial genome are associated with decreased neuronal NOS levels [437]. Alterations in skeletal muscle mitochondria are progressive [438] and could be intrinsic to skeletal muscle and not due merely to neurogenic atrophy, as assumed commonly. 

#### Human ALS and Mitochondrial-Orchestrated PCD Involving p53

PCD appears to contribute to the selective degeneration of motor neurons in human sporadic and familial ALS, albeit seemingly as a non-classical form differing from apoptosis (Figure 2 and Figure 5) [224]. Motor neurons appear to pass through sequential stages of chromatolysis (suggestive of initial axonal injury [396]), somatodendritic attrition without extensive cytoplasmic vacuolation, and then nuclear DNA fragmentation, nuclear condensation, and cell death (Figure 5) [224]. Motor neurons in people that have died from sporadic ALS and familial ALS show the same patterns of degeneration [224]. This cell death in human motor neurons is defined by genomic DNA fragmentation (determined by DNA agarose gel electrophoresis and *in situ* DNA nick-end labeling) and cell loss and is associated with accumulation of mitochondria, cytochrome *c*, and cleaved caspase-3 (Figure 5) [380,439]. p53 levels increase in vulnerable CNS regions in people with ALS, and p53 accumulates specifically in the nucleus of lower and upper motor neurons (Figure 5) [440]. This p53 is active functionally because it is phosphorylated at serine^392^ and has increased DNA binding activity [24,440]. However, the morphology of this cell death is distinct from classical apoptosis, despite the nuclear condensation [17,24,224]. Nevertheless, Bax and Bak1 protein levels are increased in mitochondria-enriched fractions of selectively vulnerable motor regions (spinal cord anterior horn and motor cortex gray matter), but not in regions unaffected by the disease (somatosensory cortex gray matter) [224]. In marked contrast, Bcl-2 protein is depleted severely in mitochondria-enriched fractions of affected regions and is sequestered in the cytosol (Figure 1) [224]. Although these western blot results lacked direct specificity for motor neuron events [224], subsequent immunohistochemistry for cleaved capsase-3 has shown numerous positive motor neurons in human ALS spinal cord (Figure 5) [439], but another study reported negative findings [441]. More recently, laser capture microdissection of motor neurons combined with mass spectroscopy-protein profiling have confirmed the presence of intact active caspase-3 in human ALS motor neurons [380]. 

These studies [24,224,380,439,441] support the concept of an aberrant re-emergence of an atypical PCD mechanism, involving p53 activation and redistributions of mitochondrial cell death proteins, participating in the pathogenesis of motor neuron degeneration in human ALS. The morphological and biochemical changes seen in human ALS are modeled robustly and faithfully at structural and molecular levels in axotomy models of motor neuron degeneration in adult mouse [442] but not in the commonly used human mutant SOD1 tg mouse models [381,439]. A role for mitochondria in human ALS pathogenesis is further supported by recent work suggesting that lithium treatment may be beneficial in ALS through mitochondrial autophagy [443].

#### Mitochondrial Pathobiology in Cell and Mouse Models of ALS

A common genetic mutation in human *SOD1* that is linked to familial ALS (Table 4) is the substitution of glycine by alanine at position 93 (G93A) [444]. SOD1 is a metalloenzyme of 153 amino acids (~16 kDa) that binds one copper ion and one zinc ion per subunit (Figure 1) [9]. This enzyme, functioning as a ~32 kDa non-covalently linked homodimer, is responsible for the detoxification and maintenance of intracellular O_2_^•-^ concentration in the low femtomolar range by catalyzing its dismutation [9,445]. SOD1 is ubiquitous (intracellular SOD concentrations are typically ~10–40 micromolar) in most tissues and possibly greater in neurons [446]. 

Data from cell culture studies reveal mitochondrial dysfunction, possibly motor neuron selective, in the presence of human mutant SOD1 (mSOD1) [447]. Expression of several mSOD1 variants increases mitochondrial O_2_^•- ^levels (Figure 1) and causes toxicity in cultured primary embryonic motor neurons [448], neuroblastoma cells [449], and NSC-34 cells (a hybrid cell line with some motor neuron-like characteristics, produced by fusion of motor neuron-enriched embryonic mouse spinal cord cells with mouse neuroblastoma cells) [450]. These responses can be attenuated by over-expression of MnSOD [449]. ALS-mSOD1 variants, compared to human wild-type SOD1, associate more with mitochondria in NSC-34 cells and appear to form cross-linked oligomers that shift the mitochondrial glutathione/oxidized glutathione ratio toward oxidation [447]. 

Gurney and colleagues were the first to develop tg mice that express human mSOD1 [451,452]. Now, these mice are used widely as an animal model of ALS [439,444]. Human mSOD1 is expressed ubiquitously in these mice by its endogenous promoter in a tissue/cell non-selective pattern against a background of normal mouse SOD1 [451]. Effects of this human mutant gene in mice are profound. Hemizygous tg mice expressing high copy number of the G93A variant of human SOD1 become completely paralyzed and die at ~16–18 weeks of age [451]. G93A-mSOD1 mice with reduced transgene copy number have a much slower disease progression and die at ~8–9 months of age [19,451]. 

Spinal motor neurons and interneurons in mice expressing G93A^high^-mSOD1 undergo prominent degeneration; about 80% of lumbar motor neurons are eliminated by end-stage disease [381,453]. Subsets of spinal interneurons degenerate before motor neurons in G93A^high^-mSOD1 [381] and some are the glycinergic Renshaw cells [453]. Unlike the degeneration of motor neurons in human ALS (Figure 5) [224], motor neurons in these mice do not degenerate with a morphology resembling any form of apoptosis [215,381,439,454]. The motor neurons degeneration seen in G93A^high^-mSOD1 mice more closely resembles a prolonged necrotic-like cell death process [381] involving early-occurring mitochondrial damage, cellular swelling, and dissolution [215,381,439,453,454]. Biochemically, the death of motor neurons in G93A^high^-mSOD1 is characterized by somal and mitochondrial swelling and formation of DNA single-strand breaks prior to DNA double-strand breaks occurring in nuclear DNA and mtDNA [381]. The motor neuron death in G93A^high^-mSOD1 mice is independent of activation of caspases-1 and -3, and also appears to be independent of capsase-8 and AIF activation within motor neurons [381]. Indeed, caspase-dependent and p53-mediated apoptosis mechanisms might be blocked actively in G93A^high^-mSOD1 mouse motor neurons, possibly by upregulation of IAPs and blockade in the nuclear import of p53 [381]. More work is needed on the cell death in G93A^low^-mSOD1 mice, because these mice could be more relevant physiologically and clinically to the human disease compared to G93A^high^-mSOD1 mouse.

Mitochondrial pathology has been implicated in the mechanisms of mouse ALS [5], but until recently, most evidence is circumstantial. In different mSOD1 mouse models of ALS, mitochondria in spinal cord neurons exhibit structural pathology [81,381,439,453,454,455,456] and some of the mitochondrial degeneration occurs very early in the course of the disease [381,454]. Mitochondrial ultrastructural microvacuolar damage in motor neurons emerges by 4 weeks of age in G93A^high^ mice [381]. It has been argued that mitochondrial damage in G93A^high^-mSOD1 mice is related to supra-normal levels of SOD1 and might not be related causally to the disease process because tg mice expressing high levels of human wild-type SOD1 show some mitochondrial pathology [457], but mitochondrial abnormalities have been found histologically also in G93A^low^-mSOD1 mice [458]. Thus, mitochondria could be primary sites of human SOD1 toxicity in tg mice irrespective of transgene copy number and expression level of human SOD1, but direct, unequivocal causal relationships have been lacking. 

Human mSOD1 proteins appear to acquire a toxic property or function, rather than having diminished O_2_^•- ^scavenging activity [459,460,461], and wild-type SOD1 can gain toxic properties through loss of Zn [448] and oxidative modification [462,463]. A gain in aberrant oxidative chemistry could contribute to the mechanisms of mitochondriopathy in G93A^high^ mice (Figure 1) [12,464]. G93A-mSOD1 has enhanced free radical-generating capacity compared to wild-type enzyme [461] and can catalyze protein oxidation by hydroxyl-like intermediates and carbonate radical (Figure 1) [465]. G93A^high^ mice have increased protein carbonyl formation in total spinal cord tissue extracts at pre-symptomatic disease [466]. Protein carbonyl formation in mitochondrial membrane-enriched fractions of spinal cord is a robust signature of incipient disease [81]. A mass spectroscopy study of G93A^high^ mice identified proteins in total spinal cord tissue extracts with greater than baseline carbonyl modification, including SOD1, translationally controlled tumor protein, and UCH-L1 [467]. Nitrated and aggregated cytochrome *c* oxidase subunit-I and α-Syn accumulate in G93A^high^ mouse spinal cord [381]. Nitrated MnSOD accumulates also in G93A^high^ mouse spinal cord [381]. Toxic properties of mSOD1 could also be mediated through protein binding or aggregation. Wild-type SOD1 and human mSOD1 associate with mitochondria [468,469]. Human SOD1 mutants associate with spinal cord mitochondria in mSOD1 mice and can bind Bcl-2 [470,471], thus potentially being decoys or dominant negative regulators of cell survival molecules (Figure 1, Table 1), but it is not known if this process is occurring specifically in motor neurons. Binding of mSOD1 (and perhaps its low-mobility species) to mitochondria has been reported to be spinal cord-selective and age-dependent [472], but this work also lacks cellular resolution. A recent biochemical *in vitro* study has shown that endogenous SOD1 in the mitochondrial intermembrane space controls cytochrome *c*-catalyzed peroxidation and that G93A-mSOD1 mediates greater ROS production in the intermembrane space compared to wild-type SOD1 [473]. Human SOD1 mutants can also shift mitochondrial redox potential when expressed in cultured cells [447]. Nevertheless, the direct links between the physicochemical changes in wild-type and mutant SOD1 and the mitochondrial functional and structural changes associated with ALS and motor neuron degeneration remain uncertain.

EM studies of motor neurons in G93A^high^ mice have shown that the OMM remains relatively intact to permit formation of mega-mitochondria [81,381]. Moreover, early in the disease of these mice, mitochondria in dendrites in spinal cord ventral horn undergo extensive cristae and matrix remodeling, while few mitochondria in motor neuron cell bodies show major structural changes [81]. Thus, the disease might start distally in mitochondria of the motor neuron processes [81,381]. Another interpretation of ultrastructural findings is that the mSOD1 causes mitochondrial degeneration by inducing OMM extension and leakage and intermembrane space expansion [474]. Mechanisms for this damage could be related to mSOD1 gaining access to the mitochondrial intermembrane space [473,474]. This mitochondrial conformation seen by EM might favor the formation of the mPTP (Figure 1); indeed, evidence for increased contact sites between the OMM and IMM in dendritic mitochondria in G93A^high^ mice has been found [81]. Another feature of motor neurons in young G93A^high^ mice, before symptoms emerge, is apparent fission of ultrastructurally normal mitochondria in cell bodies and fragmentation of abnormal mitochondria [81]. It is not clear if the cristae and matrix remodeling and the apparent fragmentation and fission mitochondria are related or independent events and if these abnormalities interfere with mitochondrial trafficking; nevertheless, morphological observations enforce the idea that mitochondria could be important to the pathobiology of mSOD1 toxicity to motor neurons in G93A^high^ mice.

The possibility of changes in mitochondrial trafficking in motor neurons of mSOD1 mice is mostly unexplored. Some data support the novel idea that mitochondria might act as messengers from distal regions of motor neurons in mSOD1 mice [5]. G93A^high^-mSOD1 mouse motor neurons accumulate mitochondria from the axon terminals and generate higher levels of O_2_^•-^, ^•^NO, and ONOO^-^ than motor neurons in tg mice expressing human wild-type SOD1 [381]. This mitochondrial accumulation occurs at a time when motor neuron cell body volume is increasing, suggestive of ongoing abnormalities with ATP production or plasma membrane Na^+^,K^+^ ATPase [381]. G93A-mSOD1 perturbs anterograde axonal transport of mitochondria in cultured primary embryonic motor neurons [475] making it possible that retrogradely transported mitochondria with toxic properties from the neuromuscular junction fail to be returned to distal processes [5,381]. Mitochondria with enhanced toxic potential from distal axons and terminals could therefore have a “Trojan horse” role in triggering degeneration of motor neurons in ALS via retrograde transport from diseased skeletal muscle. 

Motor neurons in G93A^high^-mSOD1 mice also accumulate higher levels of intracellular Ca^2+^ than motor neurons in human wild-type SOD1 tg mice [381]. The intracellular Ca^2+^ signal in motor neurons is very compartmental and mitochondrial-like in its appearance [188,381]. Abnormal elevations intracellular Ca^2+^ in G93A^high^-mSOD1 mouse motor neurons have been seen also by different Ca^2+^ detection methods [476,477]. Recent work on a mouse neuromuscular junction preparation has shown that mitochondrial Ca^2+^ accumulation is accompanied by greater IMM depolarization, specifically within motor neuron terminals of human mutant SOD1 tg mice [478]. 

NO signaling mechanisms in mitochondria (Figure 1) of ALS mice appear to be involved in the pathogenesis. Motor neurons seem to be unique regarding NO production because they express constitutively low levels of inducible NO synthase (iNOS) [188,381,402]. G93A^high^-mSOD1 mouse motor neurons accumulate nicotinamide adenine dinucleotide phosphate diaphorase and iNOS-like immunoreactivity [381,402]. iNOS is also up-regulated aberrantly in human sporadic ALS motor neurons [479]. *iNOS* gene knockout [381] and iNOS inhibition with 1400W [402] extend significantly the lifespan of G93A^high^-mSOD1 mice. Thus, mitochondrial oxidative stress, Ca^2+^ dysregulation, iNOS activation, protein nitration, and protein aggregation (not necessarily SOD1 though) are all likely intrinsic, cell automonous mechanisms in the process of motor neuron degeneration caused by mSOD1 in mice [381]. The mechanistic basis for the differences between human ALS and mSOD1 mice, regarding cell death phenotype [19,439], is not yet clear, but could be related to the extreme non-physiological expression of toxic mSOD1 or to fundamental differences in cell death mechanisms in human and mouse neurons [439] or tissue inflammation that drive motor neurons in mSOD1 tg mice to necrotic-like death according to the cell death matrix (Figure 2). Another contributing factor for this difference between human and mouse motor neurons is that mitochondria are functionally diverse and have species-specific activities and molecular compositions, including the makeup of the mPTP [480]. These possibilities allow for skepticism regarding the suitability of existing tg mSOD1 mouse lines to model human ALS.

#### The mPTP Contributes to the Disease Mechanisms of ALS in Mice

Despite the implication of toxic effects of mSOD1 on mitochondria in mouse ALS, cause-effect relationships between abnormal functioning of mitochondria and initiation and progression of disease have been uncertain. These relationships need to be known because this knowledge could lead to new mechanism-based treatments for ALS. One specific target of investigation for mitochondria in disease causality is the mPTP (Figure 1).

The mPTP was first implicated in ALS pathogenesis using pharmacological approaches. Cyclopsorine A treatment of G93A^high^ mice, delivered intracerebroventricularly or systemically to mice on a multiple drug resistance type 1a/b background, improved outcome modestly [481,482,483]. These studies were confounded by the immunosuppressant actions of cyclopsorine A through calcineurin inhibition. Pharmacological studies using CyPD inhibitors devoid of effects on calcineurin need to be done on ALS mice. Another study showed that treatment with cholest-4-en-3-one oxime (TRO19622), a drug that binds VDAC and the 18 kDa translocator protein (TSPO, or peripheral benzodiazepine receptor), improved motor performance, delayed disease onset, and extended survival of G93A^high^ mice [484]. However, another study using a different TSPO ligand (Ro-4864) did not show positive effects with G93A^high^ mice [485].

CyPD and ANT are targets of nitration in ALS mice (Figure 1) [81]. CyPD nitration is elevated in early- to mid-symptomatic stages, but declines to baseline at end-stage disease [81]. ANT nitration is pertinent because it occurs in pre-symptomatic and symptomatic stages but not at end-stage disease or in tg mice expressing human wild-type SOD1 [81]. The ANT is important in the context of age-related neurodegenerative disease because it undergoes carbonyl modification during normal aging in housefly flight muscle [486] and rat brain [487]. *In vitro* cell-free and cell experiments have shown that NO and ONOO^-^ can act directly on the ANT to induce mitochondrial permeabilization in a cyclosporine A-sensitive manner [488]. Oxidative stress enhances the binding of CyPD to ANT [489]. Some SOD1 mutants are unstable and lose copper [464,465], and interestingly, copper interactions with ANT and thiol modification of ANT can cause mPTP opening [490,491]. Together these data and future work could reveal that oxidative and nitrative damage to proteins, some of which are core components of the mPTP, in G93Ahigh mice is targeted rather than stochastic and could impinge on the functioning of the mPTP.

The role of CyPD in the process of motor neuron disease in ALS mice has been examined through gene-ablation [81]. G93A^high^-mSOD1 mice without CyPD show markedly delayed disease onset and lived significantly longer than tg mice with CyPD. The effect of CyPD deletion was much more prominent in females than in males [81]. Remarkably, female mice showed positive effects with only haplo-deletion of CyPD. *Ppif* gene ablation in tg mice with much lower levels of human mSOD1 expression and a slower disease progression (G93A^low^-mSOD1 mice) also show significantly delayed disease onset and lived significantly longer than tg mice with CyPD [81]. Thus, some form of mPTP pathobiology is occurring regardless of whether transgene expression of G93A is high or low. 

Nevertheless, most G93A-mSOD1 mice without CyPD develop eventually motor neuron disease and die. Other work on CypD null mice has shown that high concentrations of Ca^2+^ (2 mM) can still lead to mPTP activation without CyPD and that cell deaths caused by Bid, Bax, DNA damage and TNF still occur without CyPD [492]. The effects of CyPD deficiency on motor neuron cell mechanisms thus need detailed examination, but the cell death phenotype might switch or convert to another form with the attenuation of mitochondrial swelling. A switch in the cell death morphology and molecular mechanisms in motor neurons of mSOD1 mice without CyPD is an outcome consistent with the cell death matrix concept (Figure 2) [194].

## Summary and Outlook

Mitochondria have diverse functions and properties and could be critically important for the development of human adult-onset neurodegenerative disorders, including AD, PD, and ALS [7]. Structural and biochemical data from studies of human autopsy CNS as well as cell and animal models of these neurodegenerative disorders suggest that mitochondrial dysfunction is a trigger or propagator of neurodegeneration. Novel mechanisms for mitochondriopathy and neurodegeneration could involve the mPTP (Figure 1 and Figure 2). There is precedence for this logic in mouse models of AD [282], multiple sclerosis [493], stroke [494], and ALS [81]. Using ALS as one example, the mPTP actively participates in the mechanisms of motor neuron death in ALS mice in a gender-preferential pattern [81]. Thus, mPTP activation is a possible triggering event for motor neuron degeneration, and motor neuron selective vulnerability in ALS could be related to amount, composition, and trafficking of mitochondria in these cells. Further study of mitochondria in neurons, glial cells, and skeletal myocytes can define new mechanisms of cell death and disease and can lead to the identification of molecular mechanism-based therapies for treating AD, PD, and ALS.

## Abbreviations

Aβ: amyloid beta protein
AD: Alzheimer’s disease
AIF: apoptosis-inducing factor
ALS: amyotrophic lateral sclerosis
ANT: adenine nucleotide translocator
Apaf: apoptotic protease activating factor
APP: amyloid precursor protein
CNS: central nervous system
Cu/ZnSOD: copper/zinc superoxide dismutase (also SOD1)
CyPD: cyclophilin D
DISC: death-inducing signaling complex
EM: electron microscopy
ER: endoplasmic reticulum
GPe: globus pallidus external
GPi: globus pallidus internal
HtrA2: high temperature requirement protein A2
IAP: inhibitor of apoptosis protein
IMM: inner mitochondrial membrane
KA: kainic acid
LB: Lewy body
LGN: lateral geniculate nucleus
LRRK2: leucine-rich repeat kinase 2
mnSOD: manganese SOD (also SOD2)
mPT: mitochondrial permeability transition
mPTP: mitochondrial permeability transition pore
mSOD1: mutant SOD1
mtDNA: mitochondrial DNA
NAIP: neuronal apoptosis inhibitory protein
NFT: neurofibrillary tangle
NMDA: N-methy-D-aspartate
NO: nitric oxide
NOS: nitric oxide synthase
O_2_^•-^: superoxide radical
OMM: outer mitochondrial membrane
ONOO^-^: peroxynitrite
PCD: programmed cell death
PD: Parkinson’s disease
PINK1: phosphatase and tensin homolog-induced putative kinase-1
ROS: reactive oxygen species
SNc: substantia nigra compacta
Syn: α-synuclein
Tg: transgenic
TIMM: translocase of inner mitochondrial membrane
TOMM: translocase of outer mitochondrial membrane
TSPO: translocator protein 18 kDa (peripheral benzodiazepine receptor)
TNF: tumor necrosis factor
TUNEL: terminal transferase-mediated biotin-dUTP nick-end labeling
UCH-L1: ubiquitin carboxy-terminal hydrolyase-L1
VDAC: voltage-dependent anion channel
